# COVID-19 vaccine rollout: data from informal settlements in Harare, Kampala, Lilongwe and Mumbai

**DOI:** 10.1177/09562478221149876

**Published:** 2023-02-21

**Authors:** Kate Lines, Stanley Dzimadzi, Edris Lubega, Patience Mudimu-Matsangaise, Vinodkumar Rao, Junior Alves Sebbanja, Happiness Zidana, Diana Mitlin

**Keywords:** community data, COVID-19 vaccines, Harare, health equity, informal settlements, Kampala, Lilongwe, Mumbai

## Abstract

While the COVID-19 pandemic’s effect on the health of low-income urban communities in the global South has not been insignificant, the results of state responses carried out without full consideration of poverty consequences have been very serious. Vaccination, which supports both health and economic recovery, is one way people can reduce the risk of further exclusion. This field note, drawing on surveys from informal settlements in Harare, Kampala, Lilongwe and Mumbai between August and November 2021 by national affiliates of Slum Dwellers International (SDI), provides a snapshot of how global vaccine inequalities have played out across these cities. We find that access to local vaccine programmes is influenced by both global supply and existing local-level inequities; that a low or unreliable supply, among other factors, limits political will to invest in reaching already underserved communities; and that local context and the heterogeneity of communities are key to understanding low vaccine uptake.

**Figure:**
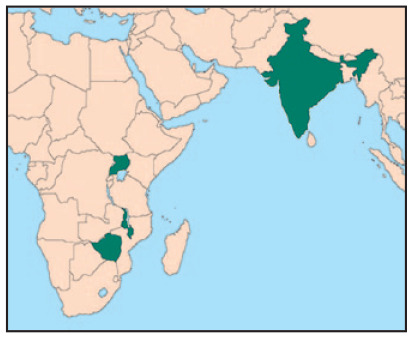


## I. Introduction

The joint issues of unequal access and low uptake of COVID-19 vaccines are still very much alive in low- and lower-middle income countries. This field note, drawing on a study from Harare, Kampala, Lilongwe and Mumbai by Slum Dwellers International (SDI) affiliates in each city, presents some grassroots perspectives on the COVID-19 vaccine rollout. By exploring how global vaccine inequalities have played out locally in 21 informal settlements across these four cities, the findings help to uncover key trends around vaccination rollout at a pivotal time. Descriptive statistics, along with qualitative evidence from residents, capture a snapshot of the situation between August and November 2021.^(1)^

## II. The COVID-19 Vaccine Context in Each Country/City

### a. Harare, Zimbabwe

**Figure 1:**
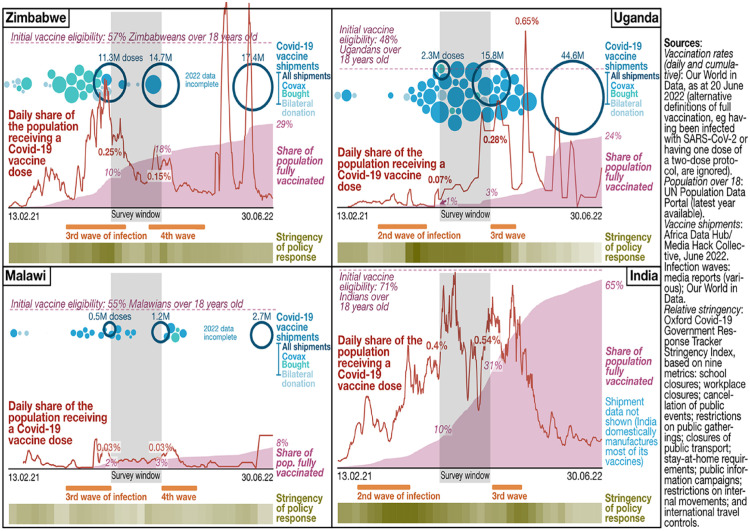
National COVID-19 vaccine context

Zimbabwe was among only 15 African countries to meet the WHO target to fully vaccinate^(2)^ 10 per cent of its population by October 2021 (Figure 1). By late August 2021, when our survey began, 10 per cent of Zimbabwe’s 15.1 million population had completed a first, full vaccination protocol, 18 per cent by late November 2021, and 29 per cent by late June 2022, when this field note was written.^(3)^

Zimbabwe’s strong relations with China have clearly contributed to its choice of COVID-19 vaccines, which are almost exclusively Chinese-made.^(4)^ Survey respondents reported Sinovac and Sinopharm as the main types available in their communities. Barring a few distribution hitches and surges in demand, vaccines have continued to be available in Harare and ongoing government campaigns have encouraged uptake.

Lockdowns and other control measures in Harare have been among the more heavy-handed in the four study cities. Vaccinations are officially voluntary, although not always experienced as such. Vaccine certificates were necessary for state employees (including health workers), and those visiting government offices, travelling on public buses, market trading, attending church and many other areas of life.

### b. Kampala, Uganda

Uganda’s vaccination drive began in earnest only towards the end of our survey window (Figure 1). In late August 2021, only one per cent of the country’s 47.1 million population had been fully vaccinated, rising to three per cent in late November 2021 and 24 per cent in June 2022. The late start was largely due to a lack of supply, even in Kampala, the capital city. Uganda has been very reliant on COVAX,^(5)^ receiving at least six types of vaccine, some shipments arriving around the same time, which generated some public confusion. Vaccines were publicly administered initially, although as supply improved, private hospitals also began to be involved (from around May 2022) in an effort to improve coverage.^(6)^

Global connections are also evident in Uganda’s vaccine supply. Media reports mention shipments of Sinovac from China, and plans to establish a Sinovac vaccine manufacturing plant in Uganda.^(7)^

When the survey began, Uganda was the only study country yet to announce universal eligibility for adults, likely related to supply constraints. Groups considered high-risk were still prioritized, including the military, health care workers, over-50s and those with underlying health conditions. Country-wide infection control measures have included two stringent lockdown periods and a night-time curfew that lasted for two years. Survey respondents also reported that vaccine certificates were at that time necessary for civil servants, public school teachers and some market vendors.

### c. Lilongwe, Malawi

Like Uganda, Malawi’s heavy reliance on COVAX for vaccine supply hugely constrained its rollout. There have been small, sporadic shipments, largely of AstraZeneca, Johnson & Johnson and Pfizer vaccines. By late August 2021, two per cent of Malawi’s 18.6 million population were fully vaccinated, three per cent by late November 2021, and still only eight per cent by late June 2022 (Figure 1).

Lockdown measures in Malawi have been relatively limited, with early government controls even temporarily blocked by the courts on the grounds of their lack of social support for low-income and vulnerable groups. Community leaders in Lilongwe reported only a few loosely enforced restrictions and vaccine mandates. Cases and deaths have reportedly been relatively low, but, as in many low-income cities, an accurate picture is constrained by limited health data collection or testing. Consequently, people have not seen vaccination as a high priority, but are more concerned with the severe associated economic crisis. Vaccine hesitancy due to misinformation appears particularly prominent in Malawi, perhaps linked to low supplies and perceived lower risks to health. Early in the vaccine rollout, Malawi incinerated 20,000 expired doses to reassure the public that the vaccines they would receive were safe.

### d. Mumbai, India

At times during the pandemic, Mumbai saw some of the highest case rates in the world. India has also imposed some of the world’s strictest lockdown measures. Mumbai is the only study city with available, reliable city-level vaccination data, and has a vaccination picture very different from those of the other cities and from India’s national rates (Figure 1): by late August 2021, 67 per cent of Mumbai’s adult^(8)^ population were vaccinated, rising to 70 per cent by late November. By April 2022, with all adults reported to be fully vaccinated, rollout was extended to school-age children and booster programmes (i.e. subsequent vaccinations after an initial full vaccination) began. At the height of the rollout, there were over 400 vaccination centres (including those run by the private sector), many in informal settlements. Three key factors underlie Mumbai’s relative success. First, government commitment, particularly municipal, to ensure universal vaccine availability; second, vocal, organized and politically well-represented low-income communities; and third, as restrictions eased, full vaccination was required to travel on suburban trains, a vital transport mode for people to reach work cheaply and quickly.

Mumbai’s inclusion in the study makes possible comparison with a relatively advanced, successful vaccine rollout in a context where half the population lives in informal settlements. Yet it is distinct in many ways: the scale of the challenge (a metropolitan population of over 20 million), strong political representation for low-income communities, and the availability of vaccines (with Indian manufacturers). Most doses from Indian manufacturers are centrally allocated to states, but during our survey, privately administered vaccines were also available for a capped fee of around US$ 9, a route open to even some informal settlement residents – often paid for by their employers to get staff quickly protected.

## III. Methodology

### a. Study locations

The survey cities, Harare, Kampala, Lilongwe and Mumbai, were selected in consultation with SDI’s secretariat. The three main criteria were:

- Major global South cities with large informal settlement populations, in countries that had begun COVID-19 vaccine rollout
- A diversity of geographical regions, city types and country income classifications
- City-based research partner organizations (all SDI country affiliates) keen to take part, with access to trusted community leaders, who are familiar with the research process

Each of the four research partners selected five or six informal settlements as study areas (Figure S1 available online) in consultation with grassroot leaders and federation members, who helped link research teams to the survey respondents. In each area, there is a strong SDI federation presence or relationship with the neighbourhood. The 21 settlements selected represent a geographic spread within each city and a range of settlement types.

### b. Survey participants

With the support of federation leaders, research teams recruited around 15 residents from each settlement (300 over the entire study) who could participate in all six fortnightly surveys. All respondents were identified as “community leaders”, defined broadly to include traditional leaders, youth leaders, community health care workers, religious leaders and SDI federation leaders – male and female and across a range of ages. We did not aim for statistical validity but rather identified a range of trusted local leaders who would have a good sense of what was going on in their communities.

### c. Data collection

Data were collected concurrently in the four cities over three months, between 26 August and 29 November 2021, using six fortnightly semi-structured questionnaires. In the fast-changing context of the pandemic, this allowed us to track changes, for instance in vaccination policy or availability, every two weeks. Data collection for each round took a week, or two (see below); the last round, which contained additional questions, took longer. Survey respondents were interviewed individually by local professionals or community-based federation data collectors, using either paper questionnaires or handheld devices. Final data entry was done online using the Qualtrics platform.

Between each round of data collection, city teams met to discuss process issues. Occasional group calls captured the local teams’ observations about changes and developments in local vaccine rollout efforts. This helped contextualize the survey data, alongside a synthesis of other (emerging) studies and news articles.

### d. Survey design

A draft questionnaire was circulated within partner organizations and to federation leaders to ensure phrasing and terminology were locally appropriate and objectives aligned with affiliates’ own. The Mumbai team translated the questionnaire into Marathi; the others were in English. Open and closed questions captured both qualitative and quantitative information. Respondents were asked both about themselves and their communities. Questionnaire and participant consent processes were tested by community leaders in each city, leading to some adjustments.

### e. COVID-19 special circumstances and ethical considerations

**Figure 2:**
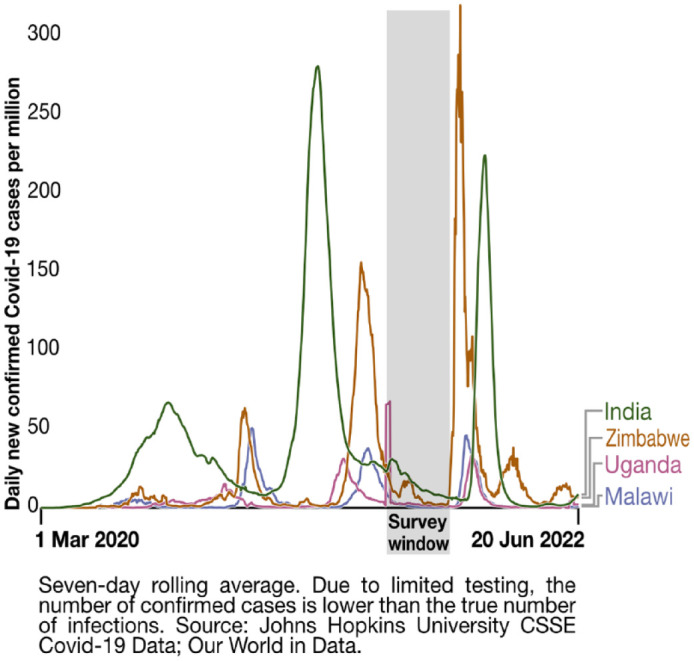
National COVID infection rates, March 2020–June 2022

Our data collection happened to take place in a relative lull between recorded waves of infection in all four study countries (Figure 2). Therefore, despite ongoing COVID restrictions, all four research partners were able to conduct at least some interviews face-to-face, observing local protocols such as social distancing and mask wearing.^(9)^ The teams considered that this resulted in greater trust and higher quality data. Most researchers used printed questionnaires within survey settlements, followed by online data entry back in the office. It quickly became clear that the process was more time-consuming than anticipated, and the window for each round was expanded from 10 days to two weeks.

Given the fast-changing pandemic and vaccine context, we were prepared to add or adjust the survey questions as data collection progressed. In the end it was only necessary to add a few follow-up questions to the last survey iteration.^(10)^ Questions on longer term socioeconomic impacts were only asked in surveys 1 and 6.^(11)^

### f. Study limitations

A small study budget of under GBP 30,000 allowed only for a snapshot in a limited time period within a volatile context. And, while we worked with well-informed, diverse leaders, we recognize that not all interest groups will have been adequately represented – for example people with disabilities. Further funding has now been secured to improve our findings by: (1) presenting our data to community groups for validation and analysis, and to local authority stakeholders; and (2) further researching the application of other approaches such as in-depth stakeholder interviews and focus group discussions, including with vulnerable groups and residents who are not leaders.

## IV. What is the COVID-19 Health Context in the 21 Study Locations?

*[We knew about a recent COVID-19 death in the settlement] from seeing the deceased person not being allowed to be put in the house for a night as is tradition.* (Community leader, F, Hatcliffe Extension, Harare, Survey round 1 [S1])

*A lot of people [in our area] might have died of COVID, but the numbers are not published.* (Youth leader, M, Hopley, Harare, S1)

*People have slacked [precautions] with reduction in cases . . . [saying] that during the hot weather COVID-19 is powerless.* (Religious leader, F, Chinsapo, Lilongwe, S6)

### a. Recent cases and deaths

Community leader respondents reported few COVID-19 cases and deaths in their areas during the survey period (Figure S2 available online). Harare settlements had the highest share of respondents who reported recent cases (on average 13 per cent of respondents) and deaths (11 per cent). In Kampala and Mumbai, fewer than five per cent of respondents reported recent cases or deaths in their area; in Lilongwe only slightly more. In all cities, reports varied considerably across settlements and survey iterations. Where the disease was known to be present, numbers were low and varied by survey between one and 21 cases and one and five deaths in a two-week period, across all 21 settlements. There are different reasons for these generally low figures, including limited testing, lack of reliable information, or an actually low disease rate. People in Harare and Lilongwe were more likely not to know about the recent health burden or local testing in their settlement; those in Kampala and Mumbai were more confident.

### b. Recent testing

Lack of data from limited testing has been key to the lower recorded COVID-19 case and death rates in many low- and middle-income countries, obscuring our understanding of how the disease burden differs in, for example, locations with a younger demographic. Excess death studies estimate COVID-19 deaths to be between nine and 16 times higher than official figures in the four study countries.^(12)^ Reliable information about cases and deaths would help local authorities to prioritize resources and communities to understand the risks they face, motivating people to take protective measures. For example, Lilongwe respondents linked declining vaccine uptake during the survey window to decreases in reported cases in their areas.

Limited testing, screening and treatment in informal settlements mean that outbreaks are less likely to be detected at an early stage and can spread rapidly. We found little testing in communities.^(13)^ The highest rates were in Harare, where a settlement average of 30 per cent of respondents said local testing was taking place; the lowest in Lilongwe (five per cent). There were fluctuations in all cities across survey iterations and between settlements. Many areas reported no testing provision, despite having a local health centre. Some Harare communities could have PCR tests at government clinics, and Mumbai communities could be screened for symptoms at hospital or special camps run by NGOs. Lilongwe and Kampala respondents mentioned a range of testing venues, including hospitals, health centres, markets and mosques, usually run by government health bodies, but had little information on the type of testing available. Notwithstanding low rates of testing, all four countries appeared to be in a relative lull between waves of infection during the survey window (Figure 2).

Apart from test results, the main sources of information on cases and deaths varied by community, and included community rumours, direct information from the family of infected people (all cities), local leaders and community health workers (Harare, Kampala and Lilongwe), and observing burial procedures that differed from the norm (Harare, Lilongwe).

## V. What Pandemic Control Measures are Currently in Place? What have been the Economic Consequences of these Measures?

*[Curfew] has done more harm than good . . . it has caused a lot of theft and poverty.* (Community leader, M, Rubaga Division, Kampala, S6)

*My business challenges are far worse and right now that’s the only urgent issue. I will go for vaccination later.* (Self-employed traditional leader, M, Mtandire, Lilongwe, S6)

State responses and shutdowns – often implemented without adequate consideration of the poverty consequences – have had serious impacts in informal settlements, where economic burdens piled upon the already substantial health burdens. Lockdowns halted much economic activity almost overnight, leaving people suddenly without ways to make a living. Lost income and employment, travel restrictions and the rising cost of staples have been major immediate drivers of impoverishment,^(14)^ and we now face the need to understand the economic impacts on communities beyond early lockdowns. There have been disproportionate effects for young people, women, migrants and informal and self-employed workers.^(15)^

In informal settlements, residents live and work in high-risk conditions, with high densities and a lack of basic infrastructure. Communities with insecure land tenure have been especially badly hit, being more likely to lack basic services and to pay higher prices for these to informal suppliers.^(16)^ In undocumented areas, it is harder for authorities to establish impacts and plan responses, and communities without legal tenure may be excluded from formal relief efforts and social safety nets, where these exist.^(17)^ Income loss or demolition can lead to eviction, worsening COVID-19 transmission risks as households resort to sharing ever more cramped spaces or migrants return to rural homes.^(18)^

### a. Employment: formal and informal workers

*[Many] informally employed people have been affected through demolished workshops, food stalls and markets.* (Vendor and community health worker, F, Hopley, Harare, S1)

*Those still working do not have as much work as they used to.* (Self-employed federation leader, F, Mtandire, Lilongwe, S6)

*Initial problems are now resolved, most are back at work.* (Casually employed community leader, F, Dharavi, Mumbai, S6)

Figure S3 (available online) shows the dramatic loss in earning opportunities in study areas for both formal and informal workers. While the situation varies, informal workers have been hit harder in almost all settlements. This is not a static situation: our findings show changes even over the short survey period. For formal workers in Harare, Kampala and (to a lesser extent) Lilongwe, things worsened between August and November; for informal workers, there was little change to an already dire situation. Mumbai was strikingly different. Community leaders reported that by late November almost everyone normally economically active was back working, in parallel with high rates of vaccination and relaxation of some restrictions for the fully vaccinated, notably local train travel.

### b. Community leaders’ own experiences

*[During the pandemic] I had access to opportunities, working to support the health ministry.* (Self-employed community health care worker, M, Mgona, Lilongwe, S6)

*I used to have two jobs, now I have one.* (Self-employed youth leader, M, Rubaga Division, Kampala, S1)

*I am a vendor, but now I sell at my gate because my market stall was demolished*. (Self-employed religious leader, F, Hopley, Harare, S1)

We asked the community leaders about their own occupations and their income over the course of the pandemic (Figure S4 available online). A vast majority (87 per cent) said their income had dropped. A smaller number, varying by city, said their income had not changed or had gone up over the pandemic (Mumbai 21 per cent; Lilongwe 16 per cent; Kampala 6 per cent; Harare 5 per cent). Self-employed people – the largest group (158 respondents) – were worst affected. Of these, 93 per cent said their income had dropped, but this was even true for 69 per cent of formally employed respondents.

### c. Savings

*This year, my savings have gone down by over half. My business is not working. My husband was retrenched [lost his employment]. The little we get every day has to serve the family.* (Self-employed federation and religious leader, F, Chinsapo, Lilongwe, S6)

*Some of our members come from other areas [so] we could not save together.* (Self-employed federation leader, F, Mbare, Harare, S1)

Networked neighbourhood women-led savings groups are a cornerstone of SDI federations of the urban poor. Their savings can boost households’ ability to meet basic needs while weathering crises. In the face of the pandemic, federations across the global SDI network demonstrated the central role that organized communities can play in responding to crises and ensuring help to those in need^(19)^ – usually in the absence of social protection. Strengthened safety nets through networks of women-led savings groups have been key to these efforts, alongside addressing basic sanitation needs, collecting data to tailor responses, and raising awareness online and in neighbourhoods. However, despite the savings safety net, many groups were badly affected by the pandemic.

**Figure 3:**
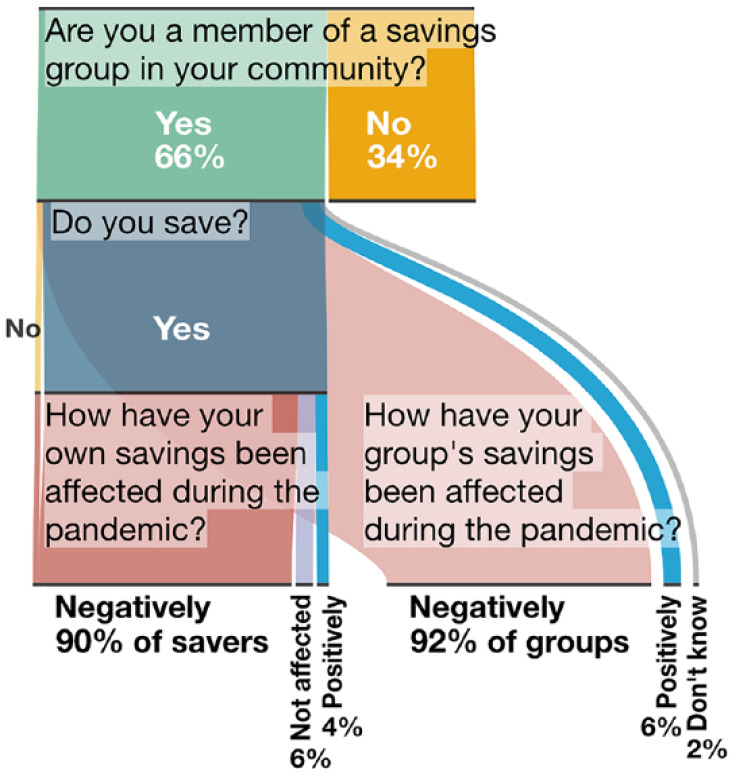
Respondents’ savings and savings groups (Lilongwe example) (survey 6 data; all-settlement average)

In the three African cities, most respondents were savings groups members. Across all cities, nearly all savers reported that both their personal and group savings had dropped. Many savings group members no longer had earnings to save (Kampala, Lilongwe). Some were still saving, but less frequently, or drawing down their savings for basic needs. Some groups had collapsed or had to recruit members from other areas (Harare). Others could no longer lend money to their members for business investments (Lilongwe) (Figure 3).

### d. Measures and restrictions during the survey period

*Many have [vaccination] because now people fear a third lockdown.* (Traditional and youth leader, F, Rubaga Division, Kampala, S6)

*There is an intense transport challenge in our settlement. People [get transport] after 6 p.m. and get home [after curfew at] 8 p.m.* (Community leader, F, Hopley, Harare, S1)

*Informally employed people [cannot] observe precautions because they sell in crowded areas.* (Self-employed federation leader, F, Hopley, Harare, S1)

Policy responses – like curfews, school/workplace closures and other measures meant to limit public exposure to disease – restrict movement, posing particular challenges to people whose livelihoods require work or travel outside “normal” hours or for long hours, including food vendors, informal traders and domestic workers. In Kampala, a curfew remained in place throughout the survey window, to be lifted only in January 2022. All Harare respondents reported lockdowns and/or curfews in August but by November these had been lifted in many settlements (curfews were later reimposed but relaxed in June 2022). Control measures were largely absent in Lilongwe and Mumbai during the survey window (Figure S5 available online).

## VI. What do the COVID-19 Vaccination Programmes Look Like from the Viewpoint of Informal Settlement Communities?

### a. The rollout

In Harare and Mumbai, almost all community leaders said public vaccination programmes were accessible to their communities (Figure S6 available online). However, some settlements in both cities experienced short-term unavailability across one or more survey iterations. In Kampala and Lilongwe in particular, reports varied by settlement and by survey round, suggesting patchier vaccine distribution and/or sparser public information. In Kampala, an average of 13 per cent of respondents said no rollout was happening; a further five per cent that the existing rollout was inaccessible to their communities, the latter group largely from one area (Makindye Division). In Lilongwe, 29 per cent of respondents to the first survey round said no rollout was happening in their city at all; this declined to an average of six per cent across the remaining surveys.

As mentioned, Zimbabwe’s and India’s vaccine supplies have been relatively consistent and abundant – albeit at times insufficient to meet demand – when compared to Uganda and Malawi. Our findings are that a low or unreliable supply negatively affects rollout and that, conversely, a reliable supply can encourage the political will to invest resources or political capital in public vaccination campaigns.

### b. Where are people getting vaccinated?

*[We] need a vaccination centre in the settlement to . . . avoid travelling long distances.* (Community health care worker, F, Stoneridge, Harare, S6)

*With a centre within our locality coordinated by the local leadership, most get it here.* (Community health care worker, M, Relocation Colonies, Mumbai, S3)

*Soldiers and nurses from our neighbourhood are vaccinated at their workplaces.* (Federation leader, F, Stoneridge, Harare, S4)

*My neighbours do their businesses outside Mtandire and so prefer to get vaccinated where they spend most of their time.* (Traditional and federation leader, F, Mtandire, Lilongwe, S3)

**Figure 4:**
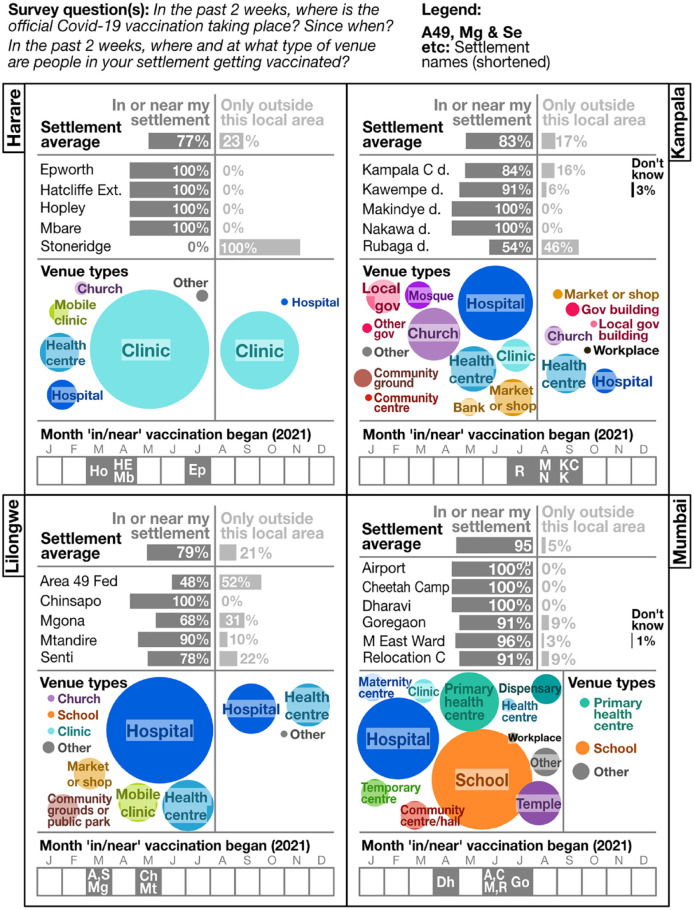
Vaccination centres: location and venues (survey 1–6 data; all-settlement average/aggregation, by city; and disaggregated by settlement)

In general, types and diversity of vaccine venues point to different distribution strategies (Figure 4). In Kampala and Mumbai, schools and churches, among other venues, were transformed into government vaccination centres. Harare relied more on existing health care sites, as did Lilongwe, where mobile vaccination vehicles were later introduced in some informal and underserved areas.

Even for informal settlements within the same city, there are big differences in proximity to vaccine centres. In Lilongwe, for example, two settlements (Chinsapo and Mtandire) are close to vaccinating public hospitals, while residents in other areas had to travel often discouragingly long distances to be vaccinated.

Many people were vaccinated at their workplace or for work-related reasons (Harare, Kampala, Mumbai), or near their workplaces for convenience (Lilongwe). Low local availability or long, off-putting queues at centres nearer home were other reasons people went further afield in all cities (Figure S7 online).

Vaccination rollout was uneven in all cities. Centres in some settlements started many months earlier than others, perhaps as low stocks limited early distribution efforts. In Mumbai and Kampala, vaccination had not begun in/near any survey settlements by March 2021, when severe second waves started. Uganda’s rollout reached the surveyed Kampala settlements between July and September 2021, as supply began to improve and eligibility was extended.

Over the course of our survey, some city authorities appeared to start addressing inaccessibility issues in parallel with wider improvements in national vaccine supply, for example introducing more mobile clinics in Lilongwe and establishing more centres nearer to communities in Kampala.

### c. Eligibility and accessibility

*There are people who come to market at dawn to sell products and sometimes end their day after 5pm. They do not have the time. Business nowadays [is hard], so it is hard to leave [early].* (Self-employed federation leader and local market leader, F, Chinsapo, Lilongwe, S6)

By early September 2021, all adults in India, Malawi and Zimbabwe were eligible for COVID vaccines. At the start of the survey, Uganda’s policy was less clear. Some groups were still being prioritized, including the over-50s, the military, health care workers and teachers. By the final survey, most said that all adults were now eligible.

**Figure 5:**
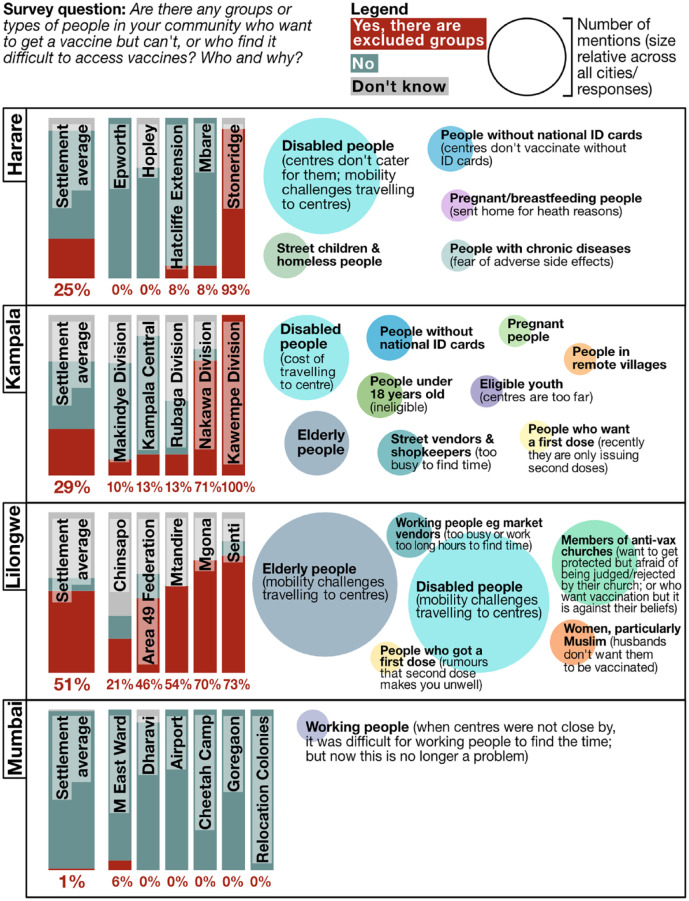
Excluded groups (survey 1–6 data; all-settlement average/aggregation by city)

Many respondents said that disabled people and elderly people faced mobility challenges in accessing clinics (Harare, Kampala and Lilongwe) which were exacerbated if centres were not nearby (Figure 5). Working people also struggled to make time to go and get vaccinated in all cities – although in Mumbai with the increase in centres this was no longer seen as a problem. Other limiting factors included: peer pressure on those in anti-vaccine churches (Lilongwe); being without national ID cards (Harare and Kampala); and women whose husbands don’t want them vaccinated (Lilongwe).

### d. Vaccine types

*The many vaccines have scared people about which one to choose.* (Federation and traditional leader, M, Kampala Central Division, Kampala, S1)

*People are looking for Sputnik. They believe it is very strong.* (Youth leader, F, Mbare, Harare, S3)

Data about available vaccine types (Figure S8 online) intersect with issues around (mis)information and understanding. For example, tendencies to prefer or reject particular vaccine types were evident in the Kampala and Lilongwe communities. Uganda and Malawi, dependent on COVAX, had perhaps less influence on which vaccines they received from the international community, at least in the early stages of rollout. Malawi has received mostly AstraZeneca and Johnson & Johnson, both for a time portrayed by Western media as especially controversial – contributing to already high levels of vaccine mistrust and misinformation. Kampala respondents spoke about the public confusion caused by the plethora of vaccine types available at different times.

### e. Second doses

*Some are willing; others have relaxed. I know people who are over 3 months after getting their first dose and have not gone for the second.* (Federation leader, F, Chinsapo, Lilongwe, S6)

In Mumbai, Harare and Kampala, second vaccines were largely available to communities, although some Harare respondents mentioned instances where second doses became unavailable in particular centres for a few weeks. In Lilongwe, Johnson & Johnson (which requires only a single dose) was the most popular and common vaccine type in use during the survey window.

Common practices for arranging second vaccines included: second dates set at first vaccination and written on a card (Harare, Kampala, Lilongwe); flexibility around (public) venue and/or both doses available at the same centres (all cities); SMS reminders (Lilongwe); mobile clinic returns to the same settlement timed with second-dose window (Lilongwe); and scheduled clinic days just for second doses (Kampala). The time required between doses varied with vaccine type, from two to three weeks in Harare for Sinovac and Sinopharm, to 12 weeks in Mumbai for Covidshield. In Harare and Kampala respondents flagged the need to bring ID, a potential barrier to those without it.

In general, community leaders reported that people in their areas who went for the first were willing to get second vaccinations. In Mumbai, restrictions on local train travel were only lifted with two-dose protection – a strong motivation to get fully covered. In Lilongwe, many respondents said people were getting *“relaxed”* and going back weeks later than the advised time. Where second-dose uptake was reportedly low/declining, contributing factors were thought to include publicly available statistics about low infection levels (Harare, Lilongwe) and misinformation about second-dose side effects (Lilongwe).

### f. Alternatives and cost to the individual

*My daughter paid a private hospital to get vaccinated, trying to avoid the long queues [at public centres].* (Community leader, F, Hopley, Harare, S1)

In Harare, Kampala and Lilongwe, COVID-19 vaccines available to residents of the surveyed areas were free. A small number of respondents in all cities said vaccines were also available privately or illegally (Harare 5 per cent, Kampala 4 per cent, Lilongwe 2 per cent). In Harare, vaccines were available in private hospitals for a capped fee of up to US$ 5, a new option intended to alleviate crowding at public centres. Only a few respondents were aware of this option – unsurprising since all study settlements are predominantly served by government health care facilities. In Lilongwe, respondents reported that vaccine certificates were widely available to the unvaccinated on the black market.

In Mumbai the situation was different. During the study, vaccines were widely available privately for a capped cost of around INR 780 (US$ 9). Indian manufacturers could sell up to 10 per cent of their product on the domestic private market and the remainder at a reduced price to the federal government, which then made allocations to states, which were widely seen as politically motivated. Demand often outstripped supply, and in our survey there were many complaints of long queues at public centres. Many respondents knew people who had been vaccinated privately, usually paid for by employers (for example, care workers). An emerging trend in Mumbai was for charitable donors, philanthropists or companies fulfilling their state-mandated corporate social responsibility responsibilities to purchase private market vaccines and donate them to local government or NGOs, for free or discounted distribution in low-income areas.

### g. Uptake numbers are inconclusive

*Our local centre gives about 200 doses every day.* (Traditional leader, M, Airport, Mumbai, S4)

Many respondents, including community health workers, could not estimate rates of vaccination uptake in their communities (Figure S9 online). In Lilongwe, 76 per cent answered “I don’t know” to the question, in Kampala 70 per cent, Mumbai 55 per cent, Harare 30 per cent – revealing a clear lack of locally-specific and publicly available statistics; although a few Mumbai respondents mentioned local vaccination numbers posted at health centres. If we look just at settlements where over half of respondents could estimate recent rates of uptake, numbers ranged widely between and within settlements: 30–500 people in Harare, 350–3000 in Mumbai (number of residents vaccinated in the past two weeks; median response per settlement across all survey iterations). Given high levels of uncertainty among respondents, the data were inadequate for tracking change over the course of the study (but see below and online Figures S10a and S10b for a discussion of the qualitative data on uptake during the survey window).

### h. Observed changes during the three-month survey window

*More people are now willing to be vaccinated after seeing there’s no side effects.* (Traditional leader, M, Nakawa Division, Kampala, S6)

*First shortage, then more vaccines were available, now most are vaccinated.* (Women’s leader, F, Relocation Colonies, Mumbai, S6)

People’s reflections on this front largely revolve around changes in uptake, accessibility and availability (Figures S10a and S10b). Overall, uptake appeared to have improved in all cities. Queues were shorter, where this was the biggest issue (Harare, Mumbai), and more and nearer centres improved accessibility for marginalized communities (Lilongwe, Kampala). However, many respondents also observed declines in vaccination uptake as the COVID threat became a lower priority with declining official case and death rates.

In Harare overall, availability appeared to have improved together with accessibility. Queues were shorter and crowds smaller at centres, and more centres were established. Uptake had correspondingly improved in four of the five settlements; however, Hopley respondents said fewer people were getting vaccinated and it was being taken less seriously.

In Kampala, the opening of more centres nearer to communities during the survey window meant improved uptake in many areas, and community leaders said people were less reluctant as they saw evidence the vaccine was safe. However, Kawempe respondents said uptake had declined. Availability improvements were noted in some areas; availability issues due to rising demand in others. There had also been changes in the type of vaccines available.

In Lilongwe, three major trends dominated the survey window. More centres, including mobile clinics, were accompanied by improved uptake. This may also relate to the rollout of a new vaccine type, Johnson & Johnson, requiring only one dose and preferred over AstraZeneca, which had more misinformation and stigma attached to it. But many Lilongwe respondents also noticed vaccination rates declining in their communities (especially Area 49 and Mgona), alongside sharp declines in officially recorded COVID-19 cases and deaths.

Changes observed in Mumbai were in line with the data elsewhere. Crowds had declined at vaccination centres, with improvements in availability and reductions in the number unvaccinated.

## VII. Attitudes to Vaccines and the Level/Nature of Vaccine Hesitancy

*Most people are going . . . in secret because of the conspiracy theories. At the same time, they want to be protected.* (Federation leader, F, Mtandire, Lilongwe, S2)

*It’s not that they don’t want it. They go to the centre and wait and waste several hours in the queue and don’t get it. They get irritated and then don’t want it.* (Traditional leader, M, Airport, Mumbai, S3)

In many low- and lower-middle income countries, initial issues with vaccine supply have gradually improved, notably after late 2021, when India began to relax its export ban and amid efforts to tackle underlying issues such as patent restrictions and the need to diversify vaccine manufacture.^(20)^ However, in many contexts this happened without proportionate rises in vaccination rates (Figure 1). Even countries less exposed to global vaccine nationalism (e.g. because less reliant on COVAX), such as Zimbabwe, have seen a levelling off in uptake.

While concerns around equitable vaccine access and country preparedness persist, vaccine confidence is another key issue, requiring culturally informed, context-specific understandings of vaccine hesitancy and misinformation.^(21)^ Some factors, although varying by context, are well documented, including under-resourced government information campaigns and the influence of social media. Others tap into underlying historical currents such as colonial-era medical abuses, or reflect local responses to perceived global-level injustices and national political dynamics around vaccine coverage.^(22)^

*Many don’t trust the government hence even the vaccine provided by it.* (Traditional leader, M, Rubaga Division, Kampala, S2)

Most community leader respondents had overwhelmingly positive attitudes to vaccines (Figure S11 online). Citywide, an average of over 85 per cent either wanted a vaccine or were fully vaccinated; a further 5–10 per cent were somewhat positive but still had concerns. More positive attitudes were articulated in terms of personal safety (all cities) and confidence in how vaccine technology works to strengthen the immune system. Social motivations included knowing people who had died of COVID (Harare); wanting to protect the health of families and communities (all cities); setting an example as a leader (Harare, Kampala, Lilongwe); and speeding up reopening and to avoid further lockdowns (Kampala, Mumbai) – allowing people to return to work, find jobs (Kampala, Lilongwe) or access areas of life controlled by vaccine mandates (e.g. public transport in Mumbai; church in Harare). A small number reflected on the influence of political leaders (Kampala) and the general importance of following government advice (Harare, Kampala, Lilongwe).

*There are many opportunities for vaccinated people*. (Youth leader, M, Mbare, Harare, S1)

**Figure 6:**
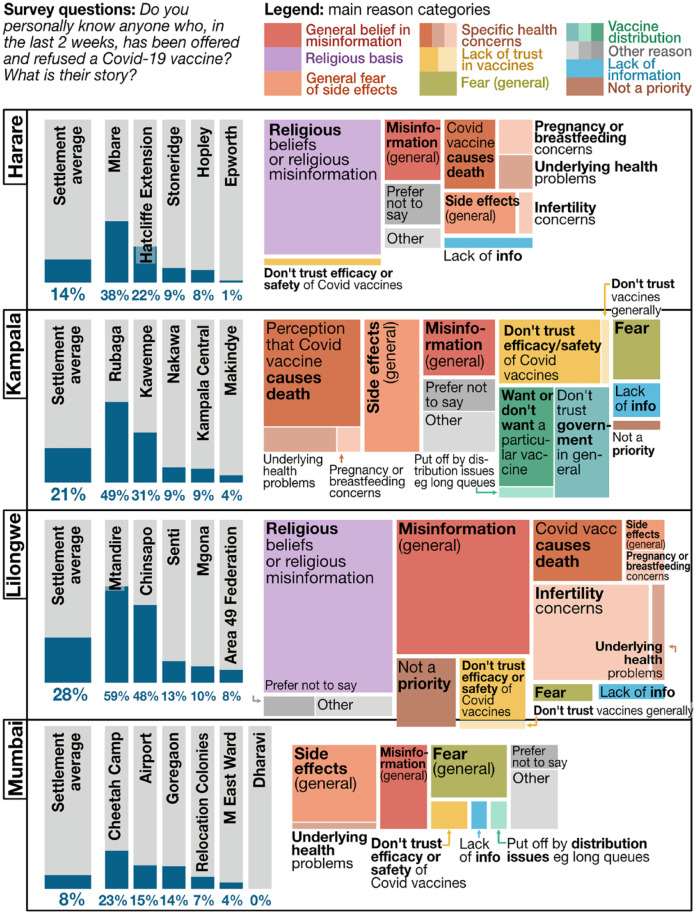
Hesitancy (survey 1–6 data; all-settlement average/aggregation, by city)

There were also anxieties,^(23)^ however (Figure 6), around vaccine safety – adverse side effects in general (Harare, Kampala) and for those with comorbidities (Harare, Kampala) and breastfeeding or pregnant^(24)^ (Kampala, Lilongwe); as well as belief in misinformation often heard via social media or from religious leaders. There was a religious basis to many people’s hesitancy (Lilongwe and Harare) ranging from personal belief that the vaccine is “satanic” to membership of churches with an anti-vax position (whether general or COVID-19-specific). Wider political dimensions of hesitancy were connected to international conspiracy theories (Lilongwe, Kampala), general vaccine scepticism (Lilongwe) and fundamental expressions of mistrust in government (Kampala).

*A friend of mine refused the vaccine because of his religious beliefs . . . He was offered a chance to get vaccinated by his employers.* (Youth leader, M, Mbare, Harare, S2)

*I am young and have a healthy body. I am not rushing to get something I am still understanding.* (Youth leader, F, Mtandire, Lilongwe, S4)

In all cities, respondents’ attitudes towards vaccines became notably more positive between the first and final surveys (Figure S11 online). This may reflect both the maturing vaccination drives in their cities (Figures S10a and S10b online) and the effects of having their attention drawn fortnightly to the issue. Mumbai respondents mentioned that many initial misconceptions and hesitancies in their communities began to dissipate once a critical mass of people had been vaccinated, providing first-hand evidence of the (lack of) severity of side effects, and the (un)reality of many misconceptions.

*There are some in the locality who are still scared of the side effects . . . but they will eventually take as it makes movement easier.* (Youth leader, M, M East Ward, Mumbai, S3)

### a. Gender lens on vaccine uptake and anxieties

*Many men have lost their jobs and are searching for employment. So, they get vaccinated . . . for potential employers.* (Federation leader, F, Chinsapo, Lilongwe, S4)

*Women are not so engaged in politics. Politics has hindered the vaccination process.* (Community leader, M, Rubaga Division, Kampala, S1)

We asked survey respondents if they had “noticed any significant difference in vaccine uptake between men and women” in their settlement (Figure S12 online). Further research could usefully build on these findings by conducting additional intersectional data collection and analysis, for example with women and men of different ages and work profiles.

Most Mumbai respondents (88 per cent) said there was no difference in uptake by gender, despite a gender gap (fewer women than men) in India’s national vaccination data at the time of the study. Such data were not available for the African countries in the study, although elsewhere South African data have shown fewer men than women getting vaccinated.^(25)^ Fewer respondents in the African cities thought there was no difference (38–47 per cent) and more respondents said more women were getting vaccinated (27–38 per cent) than those who said more men (15–21 per cent).

The findings relate differences in men’s and women’s motivations to seek vaccination to gender differences in livelihood strategies, among other factors. In all contexts, men are more likely to be formally employed, with vaccination required by existing or prospective employers. Men may travel further for work (on trains in Mumbai) or to certain public places (Kampala motorcycle taxi drivers) – both activities that require vaccine certificates. In Harare, women were more likely to work as informal traders and vendors, facing high risks of infection through contact with the public and the need to travel – and were motivated both to protect their health and by reason of being subject to occupation-related vaccine mandates. Women’s social roles were also seen to put them at greater risk of infection as they move around the community and participate in larger social gatherings (Harare, Kampala). Some respondents thought that gendered responsibilities tend to make women more aware of the need to protect their family’s health and their own (all cities), more likely to need to access government or public facilities requiring a vaccine certificate (Harare) and more familiar/comfortable in health care settings (Lilongwe^(26)^).

Respondents also discussed how opportunities to access vaccines reflect gender differences – particularly as related to the proximity of vaccine centres to where people live. Where vaccination is available in or near settlements, women’s more “flexible” time was seen to mean more opportunity to go to centres, even if there are queues (Lilongwe, Kampala). Men, more likely to work formally, are less flexible and cannot access centres operating only during working hours. They may find it easier, however, to travel further from home, for example when vaccines are only available in major hospitals (Lilongwe).

Both men and women were seen to be put off by misinformation, but for sometimes different reasons. Hesitancy was linked to men’s greater tendency to political engagement where politicians were an influential source of vaccine misinformation (Kampala). Women were thought to be concerned about side effects for breastfeeding and pregnancy (Lilongwe), and both men and women were worried by misinformation about the vaccines’ effect on fertility.

## VIII. Are People Getting the Information they Need?

*People need to know when vaccines are/aren’t available so they can plan. Sometimes they go to hospital twice and come back because there are no vaccines.* (Traditional leader, M, Mtandire, Lilongwe, S6)

*We disseminate information using megaphones, moving around the village.* (Community leader, F, Rubaga Division, Kampala, S1)

**Figure 7:**
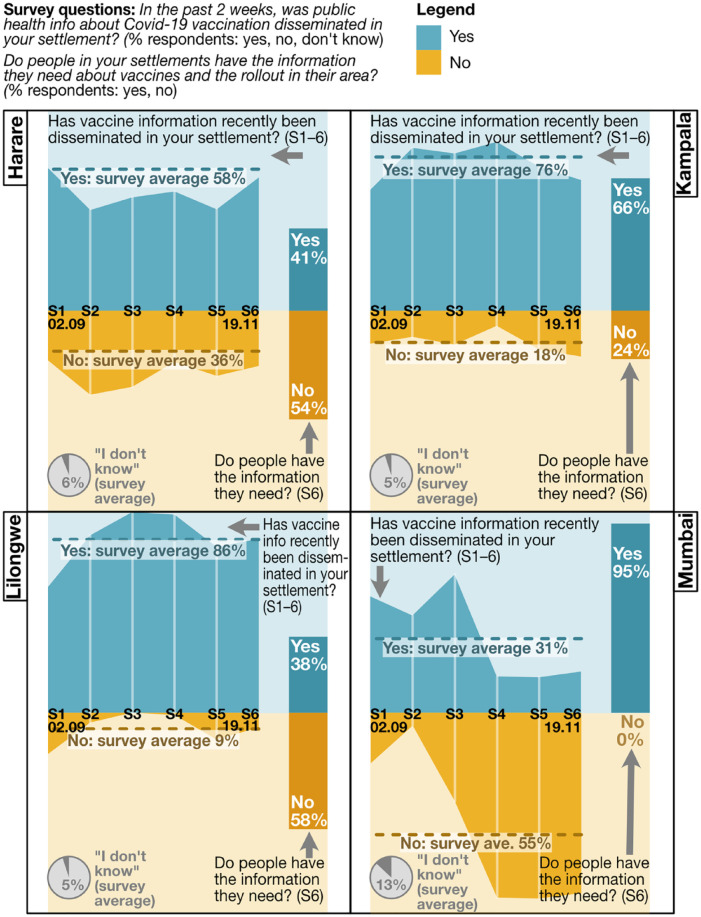
Information (survey 1–6 data; survey 6 data; all-settlement average/aggregation, by city)

Many community leaders in the African cities said COVID-19 vaccine/vaccination information had recently been disseminated in their settlements (58 per cent Harare, 76 per cent Kampala, 86 per cent Lilongwe). Across cities and settlements, modes of dissemination ranged from TV and social media to local radio, community gatherings and in-settlement door-to-door campaigns and use of public address systems. Agents included national and city governments, local and international NGOs, local leaders and community health workers.^(27)^ However, fewer survey respondents thought their communities “had the information they needed” about vaccines and the rollout in their area^(28)^ (41 per cent in Harare, 66 per cent Kampala, 38 per cent in Lilongwe) (Figure 7).

Diverse communities living in urban informal settlements represent a range of information needs. Studies have found vaccine uptake lower among marginalized groups with less access to the technology needed for reliable information about availability, venues, safety and side effects, or to book appointments.^(29)^ Good, accessible information on these fronts, lacking in many survey areas, can create trust and save communities time, especially in contexts of low/unreliable national supply or distribution challenges. Waiting in long queues or travelling long distances, only to find vaccines unavailable, were intense sources of frustration (all cities), especially for the self-employed and people working late/long hours. In Stoneridge, Harare, with no nearby health centre, many respondents flagged the need for better information (as well as nearer vaccinating centres). People also needed a better understanding of the types of vaccine available (Harare, Kampala). And in Lilongwe, where the gap was largest between provision of information and its perceived adequacy, many respondents highlighted a need for information from (importantly) sources trusted by communities, to counter specific disinformation and misinformation.

In Mumbai, almost everyone thought communities had the information they needed: most people were vaccinated, and good uptake was evidence of adequate information. Communities shared experiences and information, centres displayed vaccine availability and medical workers provided people with advice about side effects and medicines to counter them.

Information dissemination and adequacy varied by settlement and changed over the course of the survey window. In Harare, respondents in Epworth and Hopley largely thought the public health information being provided was adequate, but those in Hatcliffe Extension, Mbare and Stoneridge did not. In many Kampala settlements, NGOs and village health teams were leading awareness-raising campaigns, sometimes door-to-door; in others, respondents flagged the need to better involve local leaders.

Local-level information sources were seen to be key in Kampala, particularly village health teams, and in Lilongwe, where the Ministry of Health reaches out to communities via local leaders. In Mumbai, dominant sources were municipal authorities and primary health care workers, although local leaders played a key role in early stages of the rollout. In Harare, community health workers and local clinics also provided information.

## IX. Conclusions

*People are struggling to make ends meet. While there is much talk about vaccines, there should also be effort to help people be [financially] resilient in the face of COVID-19.* (Traditional leader, M, Mtandire, Lilongwe, S6)

Since our data collection ended, global COVID-19 vaccine inequalities have substantially lessened, although unequal access and low uptake are still concerns, and lagging demand and persistent public hesitancy remain legacies of earlier failures. Societies are still threatened by the possibility of new variants, which are most likely to emerge where there are high rates of infection and low rates of vaccination.

Informal settlement communities have been hit disproportionately hard, and existing inequalities, for example in health care provision, can have a multiplier effect. Different groups have been affected differently and access to vaccines is only one among many necessary measures for health, social and economic recovery. Across many contexts, older citizens and those with comorbidities are still very nervous about COVID-19 outbreaks. And, while younger people may now be less worried by health risks – focusing instead on the now-compounded socioeconomic challenges – all are aware of the ongoing threat of further state responses and shutdowns implemented without adequate consideration of poverty consequences, the results of which have already been very serious.

Multiple lessons can be drawn for future pandemics from the findings, specifically around vaccine rollout, and for pandemic responses more generally. A recognition of the significance of the local context is all important.

To improve pandemic response and recovery strategies, governments need to understand how vaccine campaigns can better reach underserved urban communities – not least because dense informal settlements with poor provision of basic services pose an ongoing infection risk. They also need to assess the effects of policy measures to understand how low-income communities can best be supported to recover and build resilience. State social protection measures could potentially be built around existing community-level savings systems. Recognition must be given to the penalizing effect of vaccine mandates for low-income and informal workers, who are likely to live in areas with poorer access to vaccine centres and yet lack the time and means to travel to get vaccinated further away – especially in situations where national supply and local availability are limited. Attention must also be given to improving the local availability of vaccines, especially important as a more effective way to reach women.

There are also important roles for community leaders and settlement-level information networks that must be recognized. Community-level health professionals should be prioritized, with access to information and specialized training in pandemic preparedness and post-pandemic realities. These local leaders and professionals are best placed to understand local information needs and respond to the context-specific nature of hesitancies and vaccine anxieties. Community data collection, among other ways to capture community knowledge, is also critical to the improved understandings that underpin good policy, and strengthens existing community systems, such as SDI networks.

## Supplemental Material

sj-pdf-1-eau-10.1177_09562478221149876 – Supplemental material for COVID-19 vaccine rollout: data from informal settlements in Harare, Kampala, Lilongwe and Mumbai Click here for additional data file. Supplemental material, sj-pdf-1-eau-10.1177_09562478221149876 for COVID-19 vaccine rollout: data from informal settlements in Harare, Kampala, Lilongwe and Mumbai by Kate Lines, Stanley Dzimadzi, Edris Lubega, Patience Mudimu-Matsangaise, Vinodkumar Rao, Junior Alves Sebbanja, Happiness Zidana and Diana Mitlin in Environment & Urbanization

## Footnotes

1. For broader discussion of how we understand global and local COVID-19 vaccine inequalities please refer to Lines et al. (2022b); Mitlin and Lines (2022); for a fuller report of the survey data findings, including settlement-level disaggregation, see Lines et al. (2022a).

2. “Fully vaccinate” means a full course not including boosters.

3. All statistics from Our World in Data n.d., as at 23 August 2022.

4. These were first purchased, later received through COVAX.

5. COVID-19 Vaccines Global Access (COVAX) is the global mechanism for equitably sharing COVID-19 vaccine doses. Administered by the World Health Organization, it relied heavily on largely inadequate donations of funds and doses by rich countries. Widespread vaccine nationalism also undermined COVAX’s global solidarity efforts from the start. India’s mid-2021 decision to halt vaccine exports for several months was a further setback, curtailing COVAX’s access to the Indian manufacturers on which it was overly reliant.

6. See https://www.theeastafrican.co.ke/tea/science-health/uganda-allows-private-hospitals-to-charge-for-covid-vaccines-3814170.

7. See https://www.monitor.co.ug/uganda/news/national/museveni-meets-sinovac-vaccine-manufacturers-3826444; Sinovac Biotech is a Chinese company authorized by the World Health Organization to manufacture and distribute COVID-19 vaccines.

8. Note different unit of measurement to the country statistics above.

9. In the few instances face-to-face was not possible, interviews were done over the phone.

10. See annex in Lines et al. (2022a).

11. How to read the data visualizations: each full-page data visualisation contains the following information to help the reader: the survey questions to which the data relates; the data analysed and how it is disaggregated; a legend. All visualizations disaggregate by city and some by settlement and/or survey. For visual simplicity, where survey dates are shown these are labelled with the midpoint of each of the six survey iteration windows.

12. See https://www.economist.com/graphic-detail/coronavirus-excess-deaths-estimates

13. We only asked respondents about the availability of nearby testing.

14. Lenhardt (2021).

15. ILO (2021).

16. Corburn et al. (2020).

17. Cities Alliance (2021).

18. Dupraz-Dobias (2020).

19. Cities Alliance (2021).

20. Schiffling (2021).

21. Africa Centres for Disease Control and Prevention (2021); Samarasekera (2021).

22. Mutombo et al. (2022).

23. For this analysis, we drew on the Institute of Development Studies’ vaccine anxieties framework described in Leach et al. (2022), which places “vaccine anxieties” on a spectrum from positive perspectives (confidence, desire to get vaccinated) to negative (hesitancy, concern) and encompasses three dimensions: bodily vaccine anxieties; social dimensions; and wider political experiences.

24. Safety concerns are not necessarily related to consumption of misinformation. Initially, many countries only recommended COVID vaccination for pregnant women who were “clinically vulnerable”. At the time our study took place, scientific evidence and official advice on safety for pregnant and breastfeeding women were still evolving.

25. Gibbs (2021).

26. This has also been observed in research in South Africa.

27. For a fuller picture of city-level landscapes of information dissemination (kinds of information, modes and agents), see the Figure 27 data visualisations in Lines et al. (2022a).

28. Only asked in survey 6.

29. Badiuzzaman and Akter (2021).
