# Supplementary material for: COVID-19 vaccine rollout: data from informal settlements in Harare, Kampala, Lilongwe and Mumbai
Source: Environ Urban. 2023 Feb 21;35(1):49–73. doi: 10.1177/09562478221149876 (PMC9947425; doi:10.1177/09562478221149876)

Figure S7: Vaccination outside settlement (survey 1–6 data; all-settlement average/aggregation, by city)

Survey question: *In the past 2 weeks, do you know of anyone living in your settlement who has received a vaccine outside of the local area? Can you tell us about them and their vaccination(s)?*

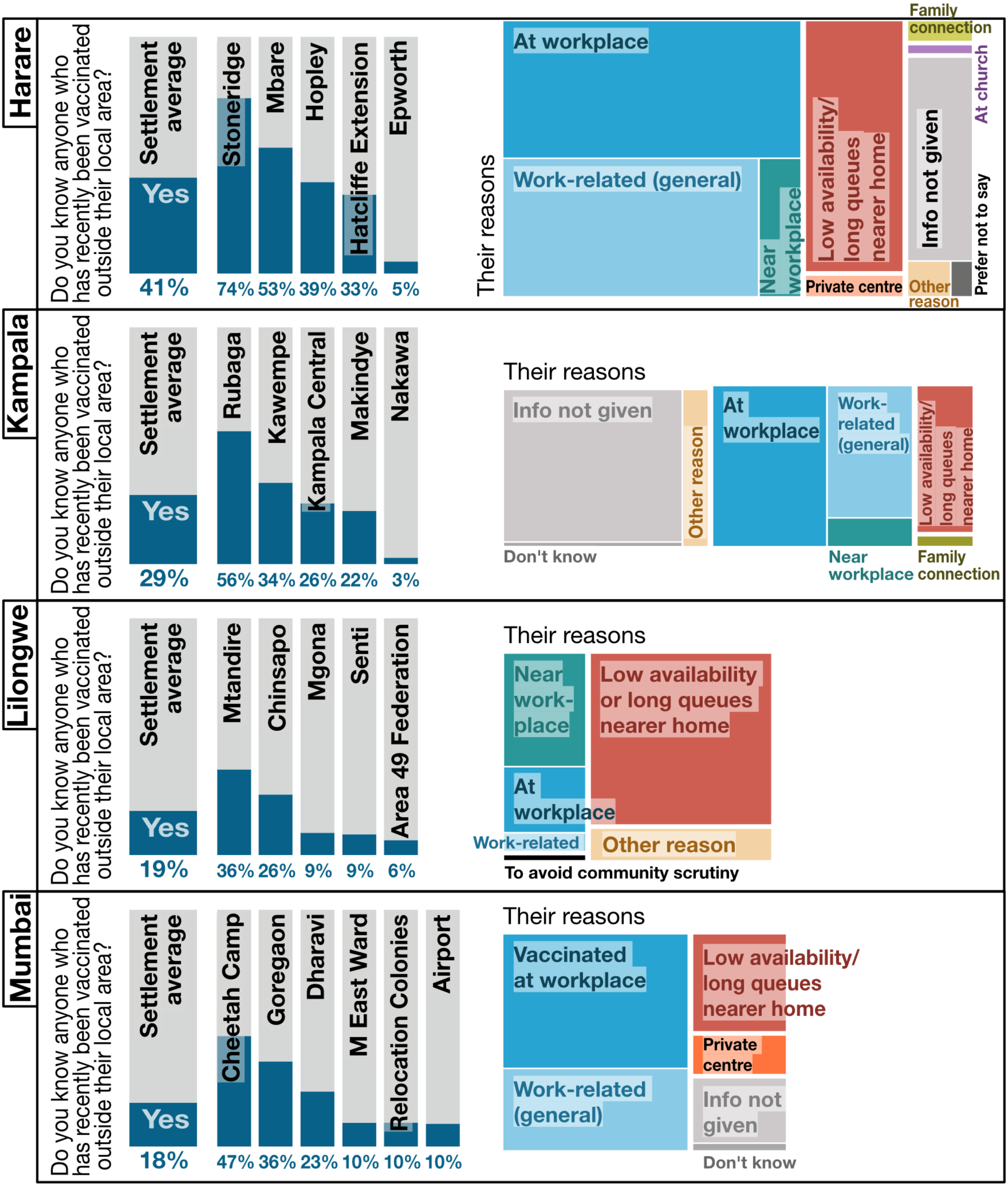

Figure S8: Vaccine types available (survey 1–6 data; all-settlement average, by city)

Survey question: *In the last 2 weeks, which vaccines are people who live in your settlement being offered? Select all that apply.* Percentage respondents mentioning a particular vaccine.

Notes: Weighted average by mentions of type per respondent (excluding "don't knows"). City average number of vaccine types mentioned per respondent: Harare = **1.9** Kampala = **1.6** Lilongwe = **1.4** Mumbai = **1.1**

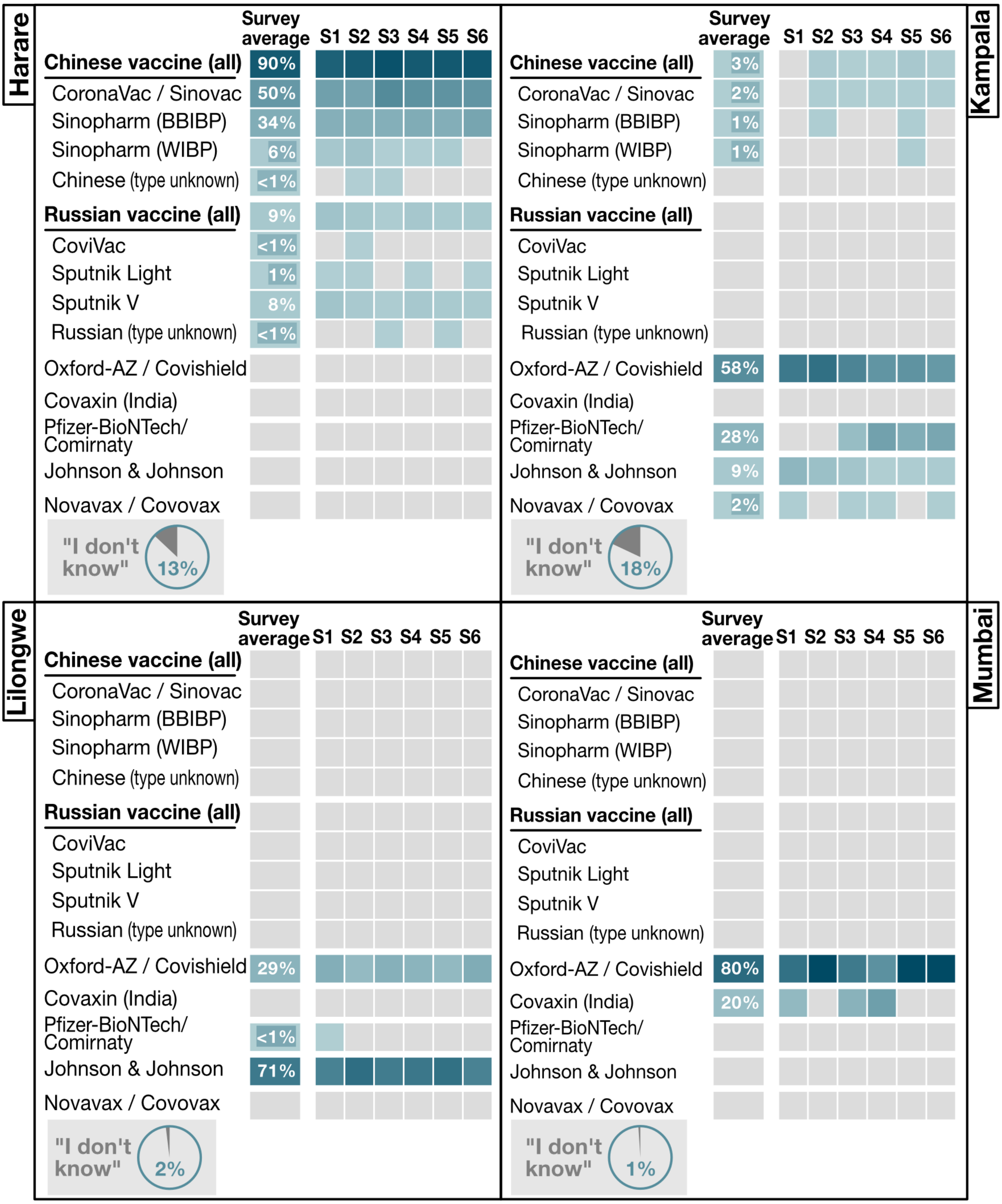

Figure S9: Numbers of people getting vaccinated (survey 1–6 data; all-survey median and interquartile range, by city and settlement)

Survey question: *Over the last 2 weeks, roughly how many people who live in your settlement have got a Covid-19 vaccine?*

Notes: Many people said "I don't know", therefore insufficient data to track change over time. India visualised at 50% scale relative to other cities. Ordered by median response, largest to smallest.

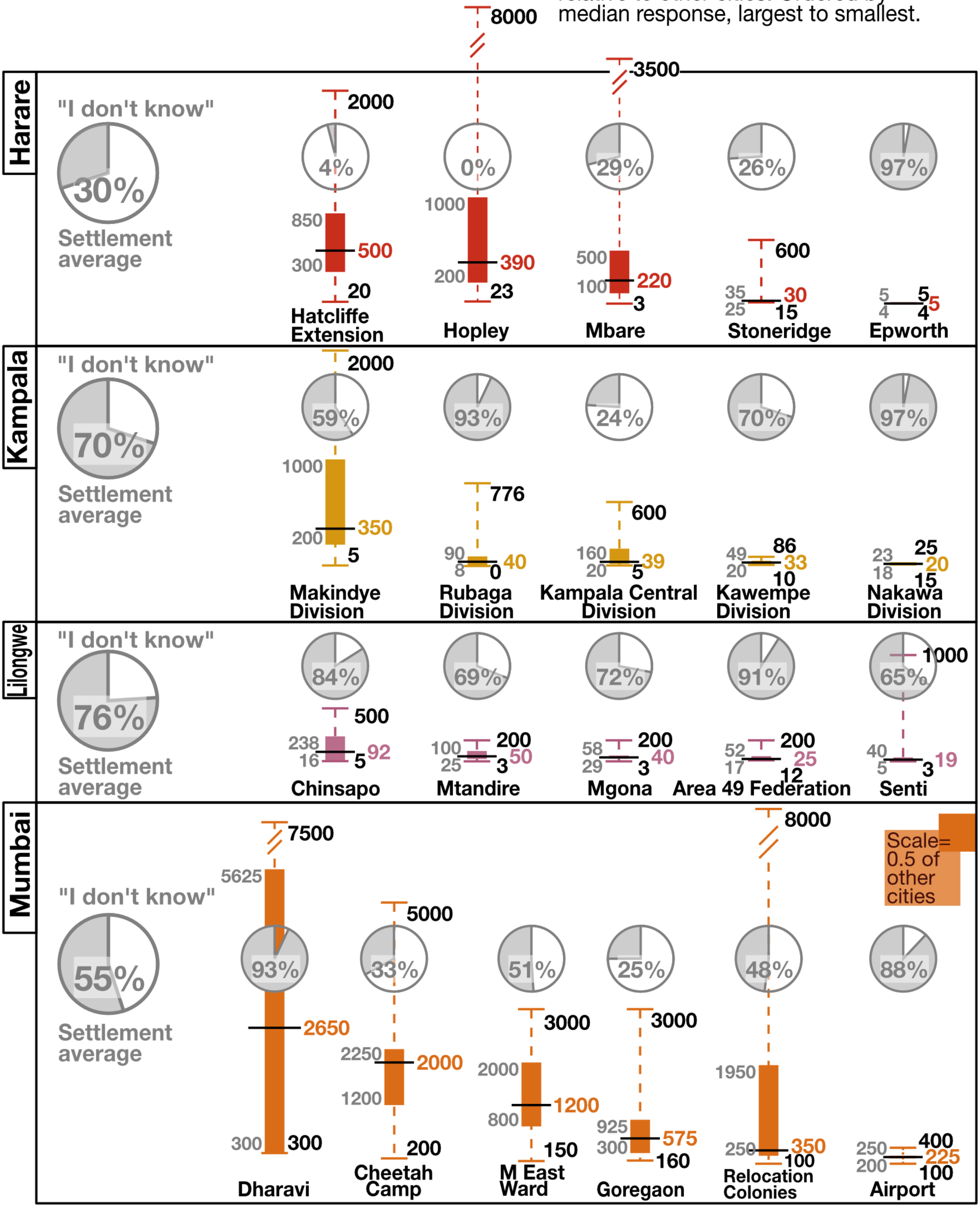

Figure S10a: Changes over the survey period (survey 6 data; A–Z by frequency of mention; excludes single mention issues; aggregated by city and settlement)

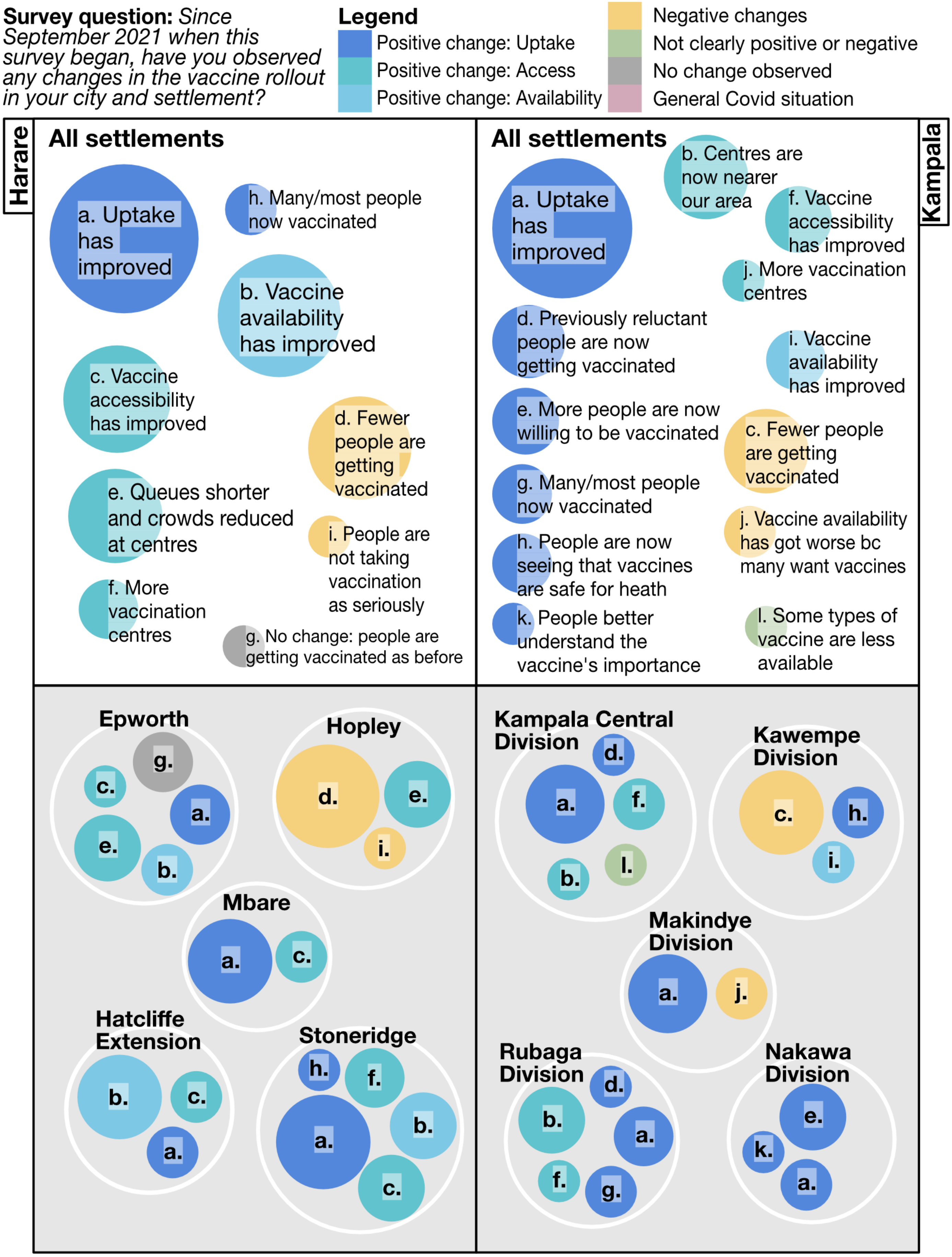

Figure S10b: Changes over the survey period (survey 6 data; A–Z by frequency of mention, city-level; excludes single mention issues; aggregated by city and settlement)

Survey question: *Since September 2021 when this survey began, have you observed any changes in the vaccine rollout in your city and settlement?*

Legend

- Positive change: Uptake
- Positive change: Access
- Positive change: Availability
- Negative changes
- Not clearly positive or negative
- No change observed
- General Covid situation

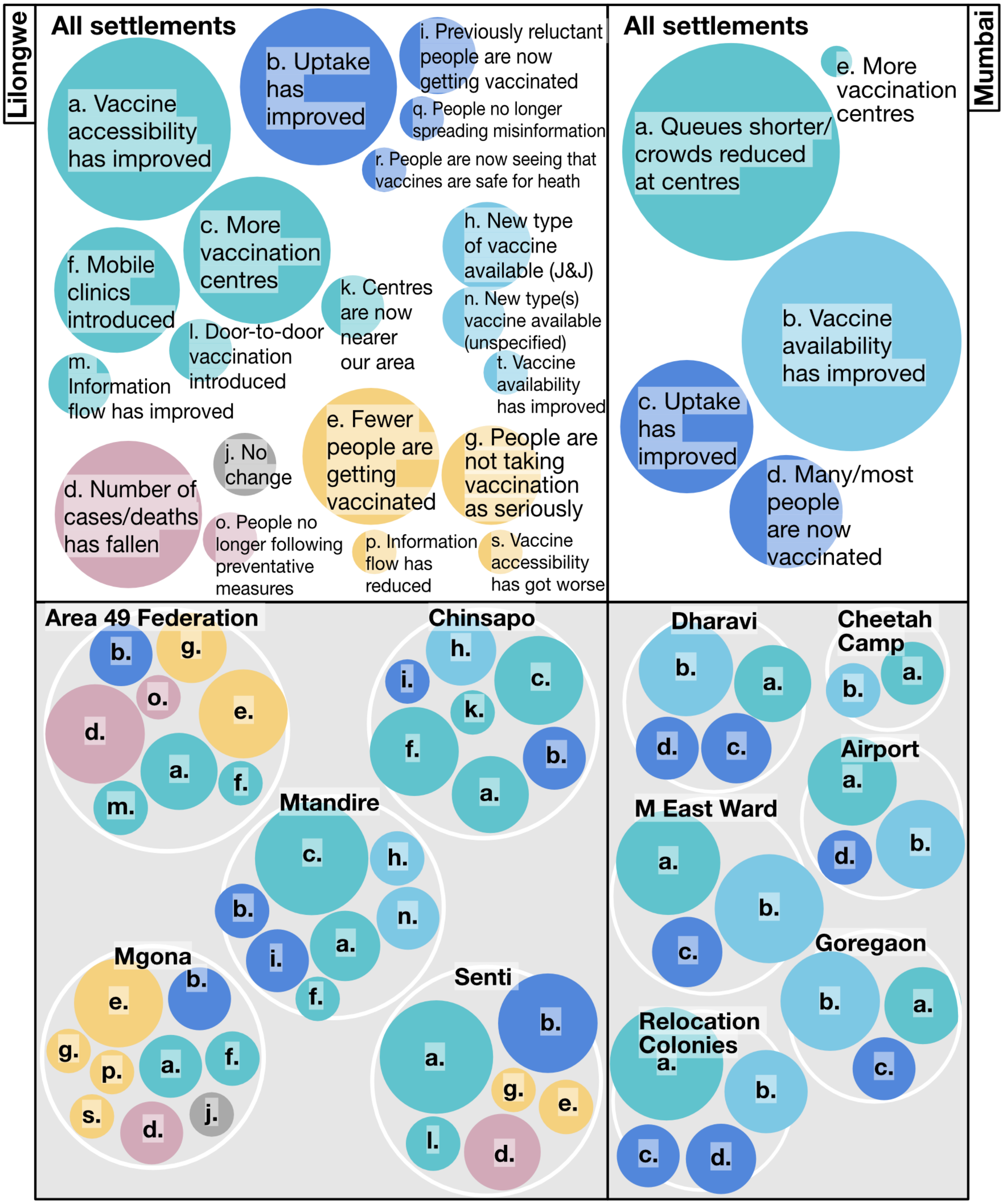

Figure S11: Respondents' attitudes (survey 1–6 data; all-settlement average/aggregation, by city)

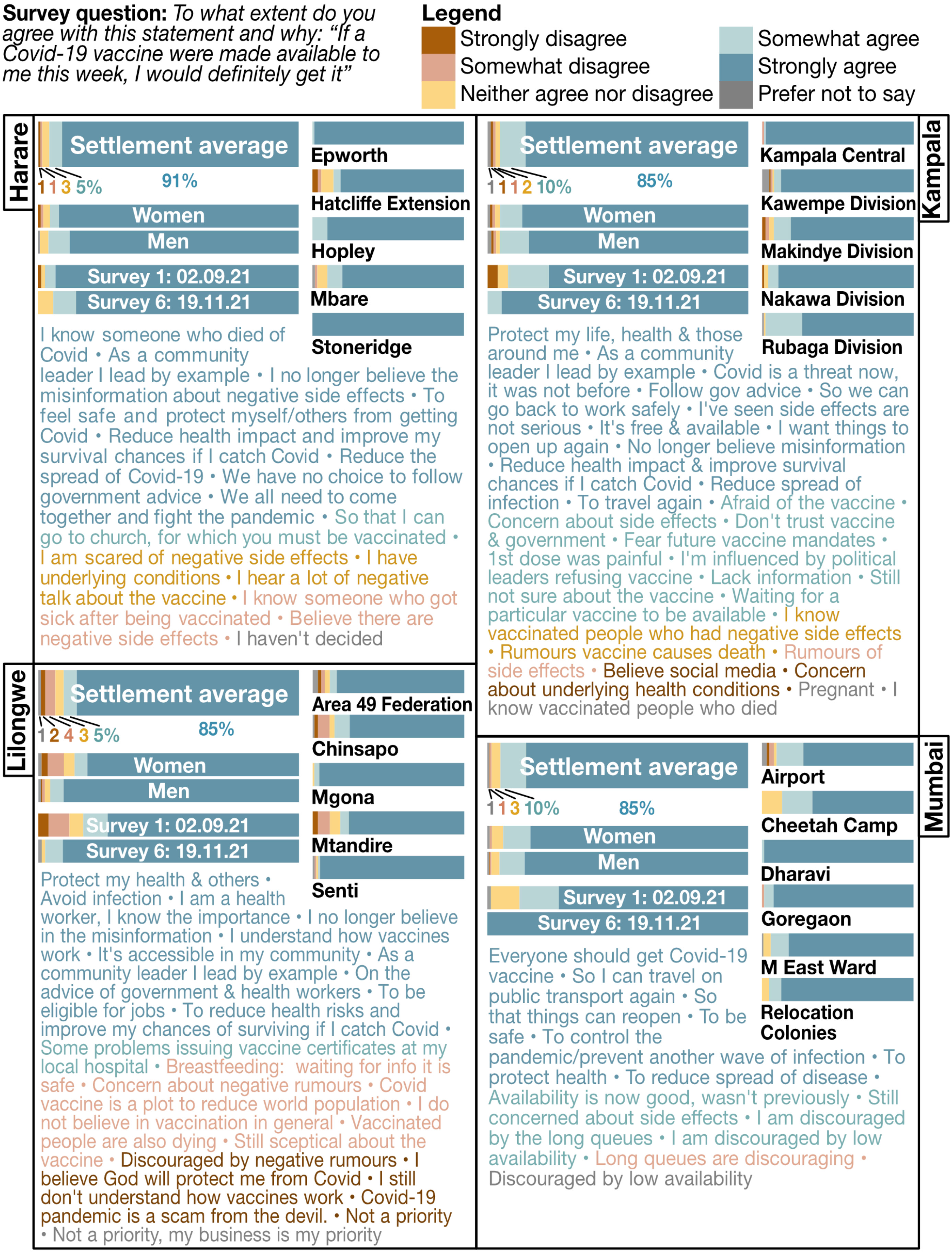

Figure S12: Gender differences in vaccine uptake (survey 1–6 data; all-settlement average, by city)

Survey questions: *Have you noticed any significant difference in vaccine uptake between men and women who live in your settlement?*

Notes: Analysis by respondents' gender revealed a very slight bias towards their own gender.

Legend

- More women than men are getting vaccinated
- More men than women are getting vaccinated
- No difference observed

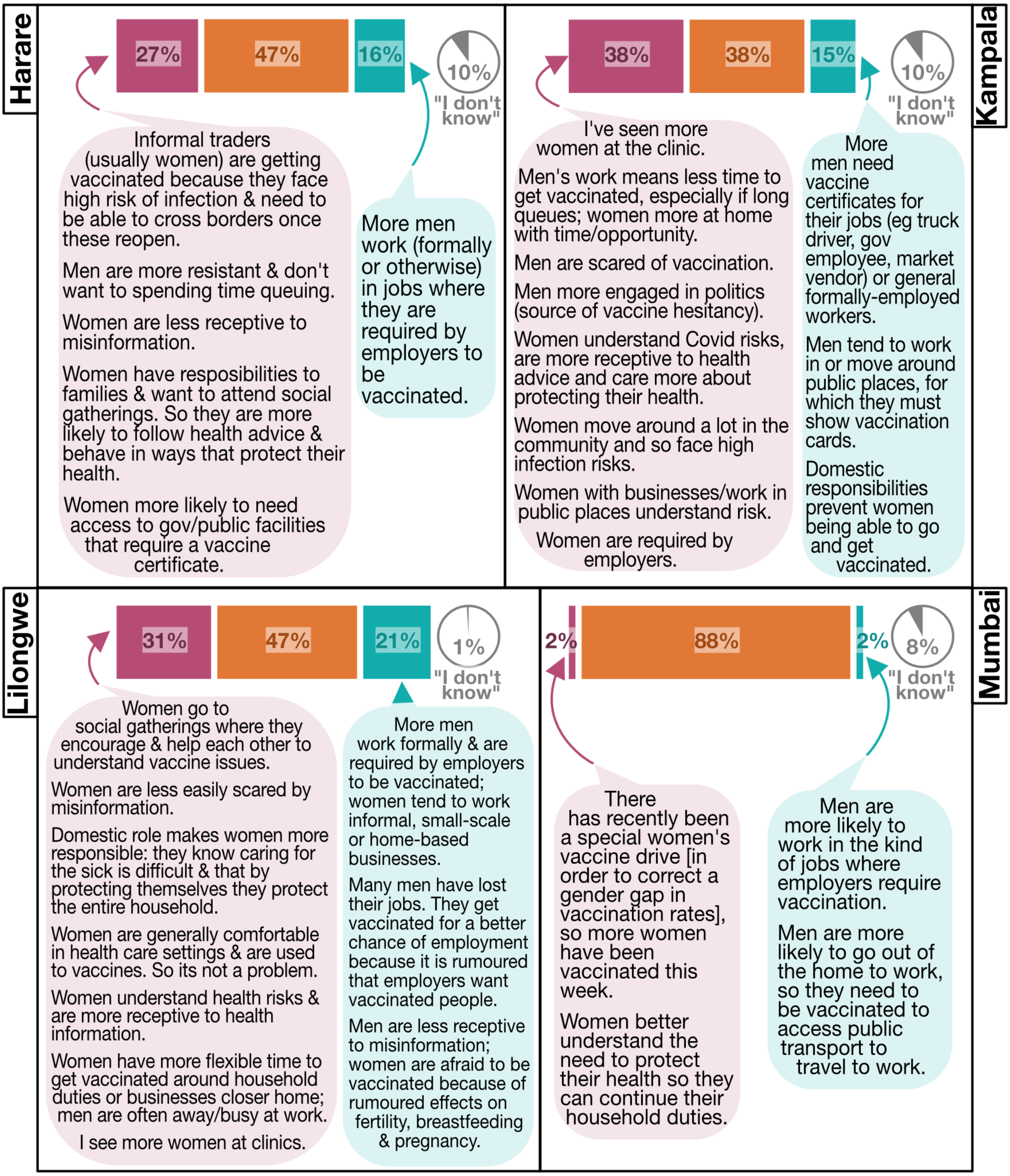

Figure S1: Maps showing survey areas

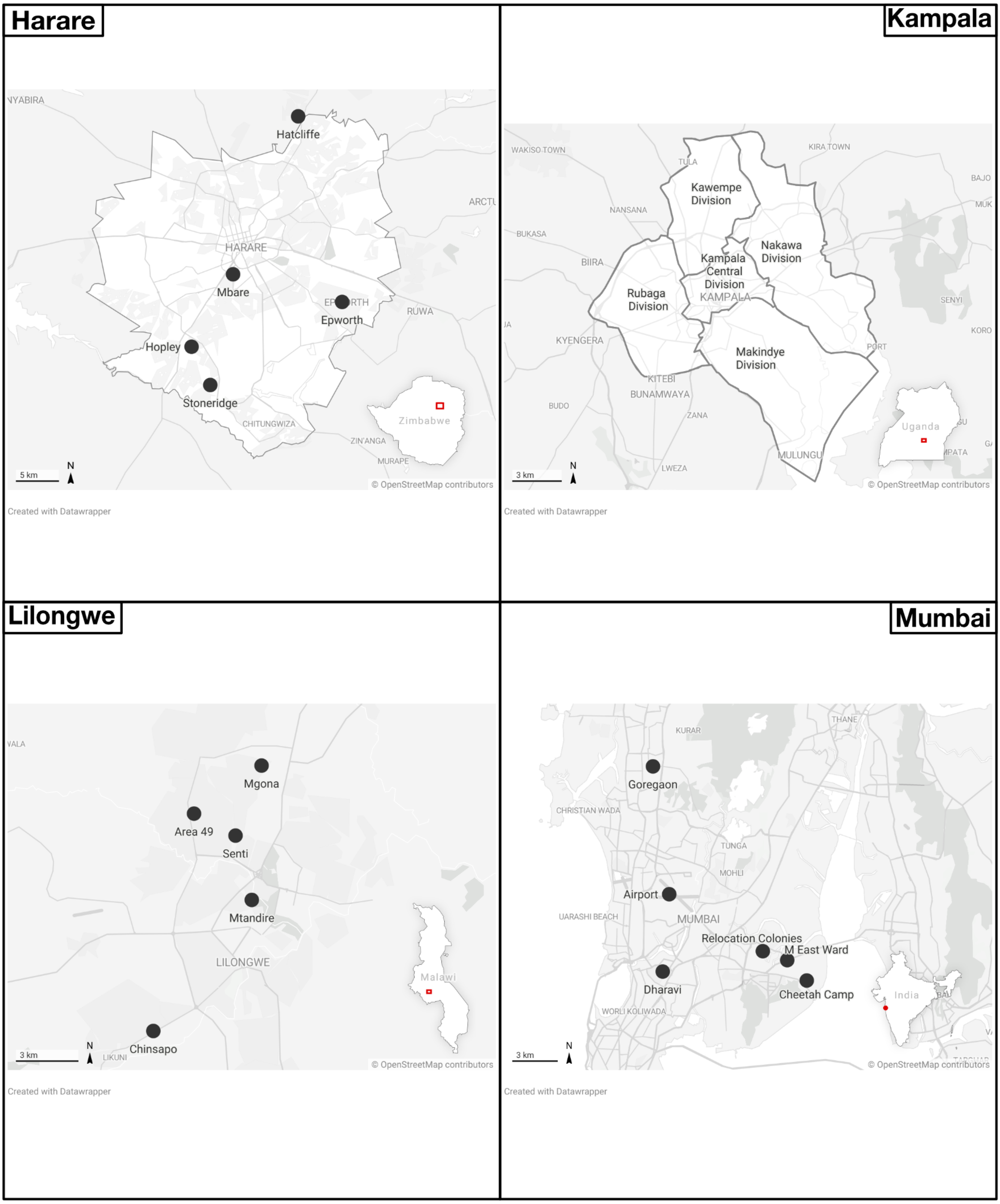

Figure S2: Recent Covid cases and deaths in the settlements (survey 1–6 data; all-settlement average, by city; disaggregated by settlement)

Survey question: *In the past 2 weeks, have there been any (reliable) reports of new (1) infections or (2) deaths of Covid-19 in your settlement? Approximately how many new (1) cases or (2) deaths have there been in your settlement in the past 2 weeks?*

Legend

Average % respondents reporting any **cases** or **deaths** in their settlement in the past 2 weeks (across all surveys)

One reported **case** or **death**

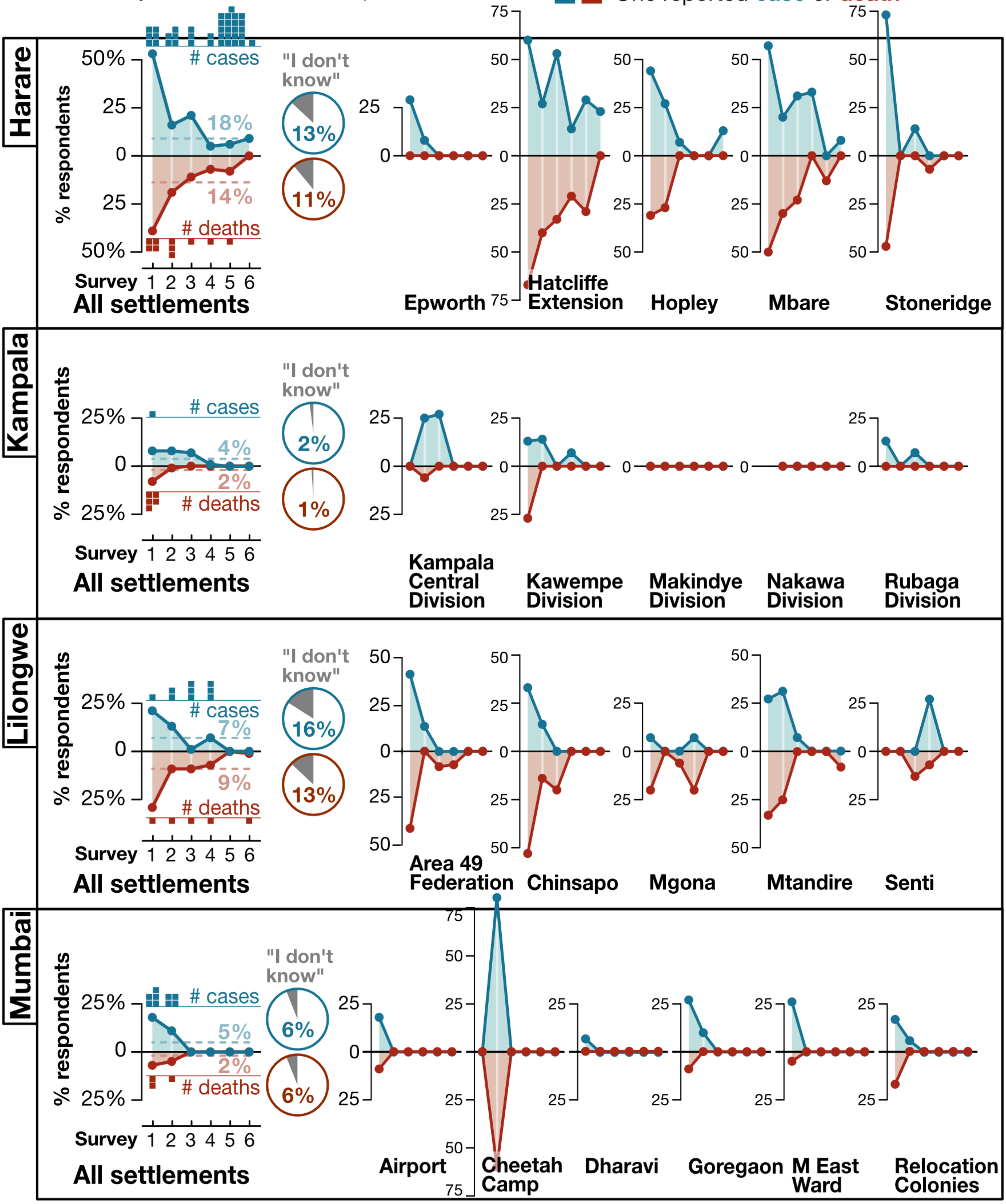

Figure S3: Impact on work and employment (informal and formal workers) (survey 1 and 6 data; all-settlement average, by city; disaggregated by settlement)

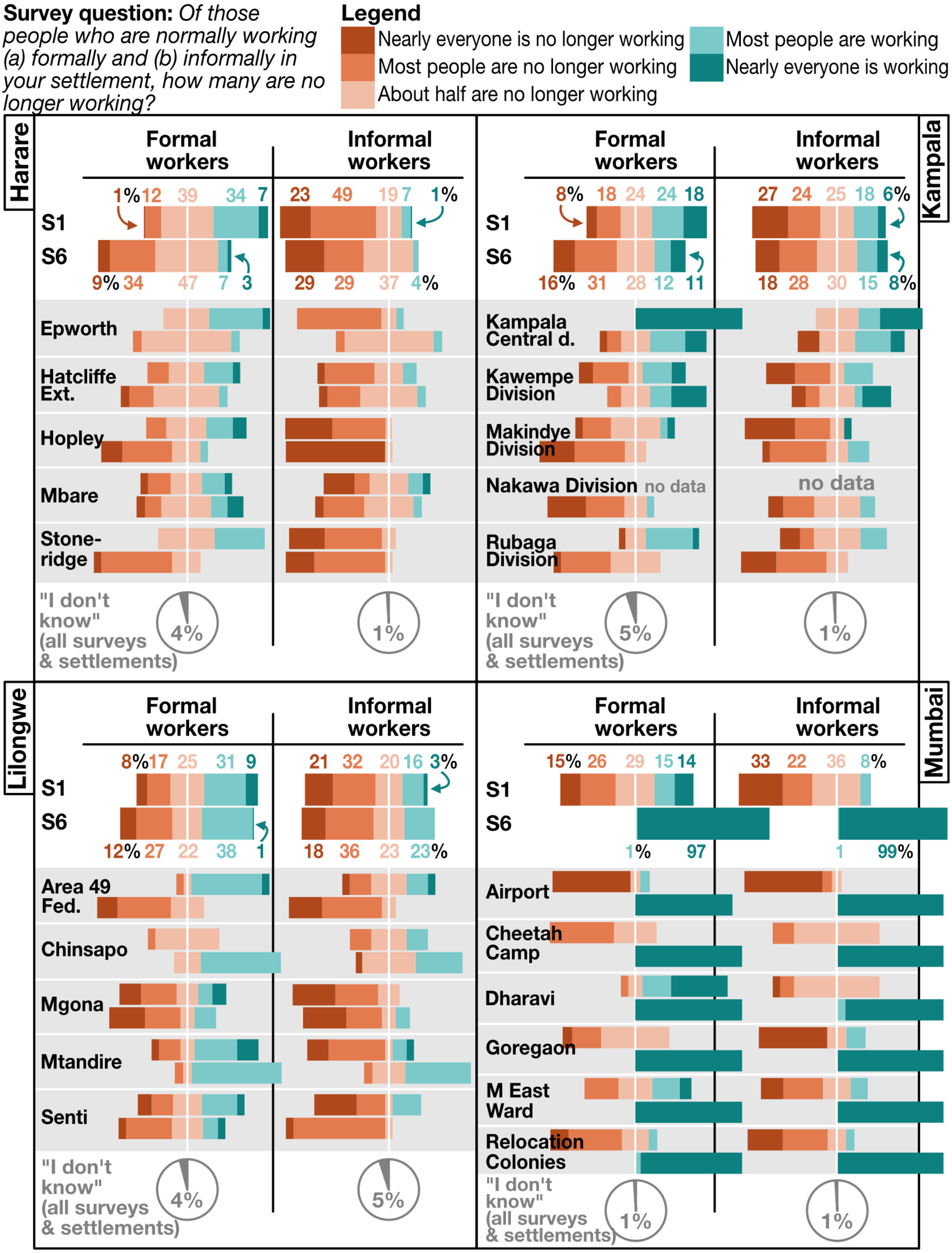

Figure S4: Respondents' occupations and income impact (survey 1 and 6 data; all-settlement average, by city)

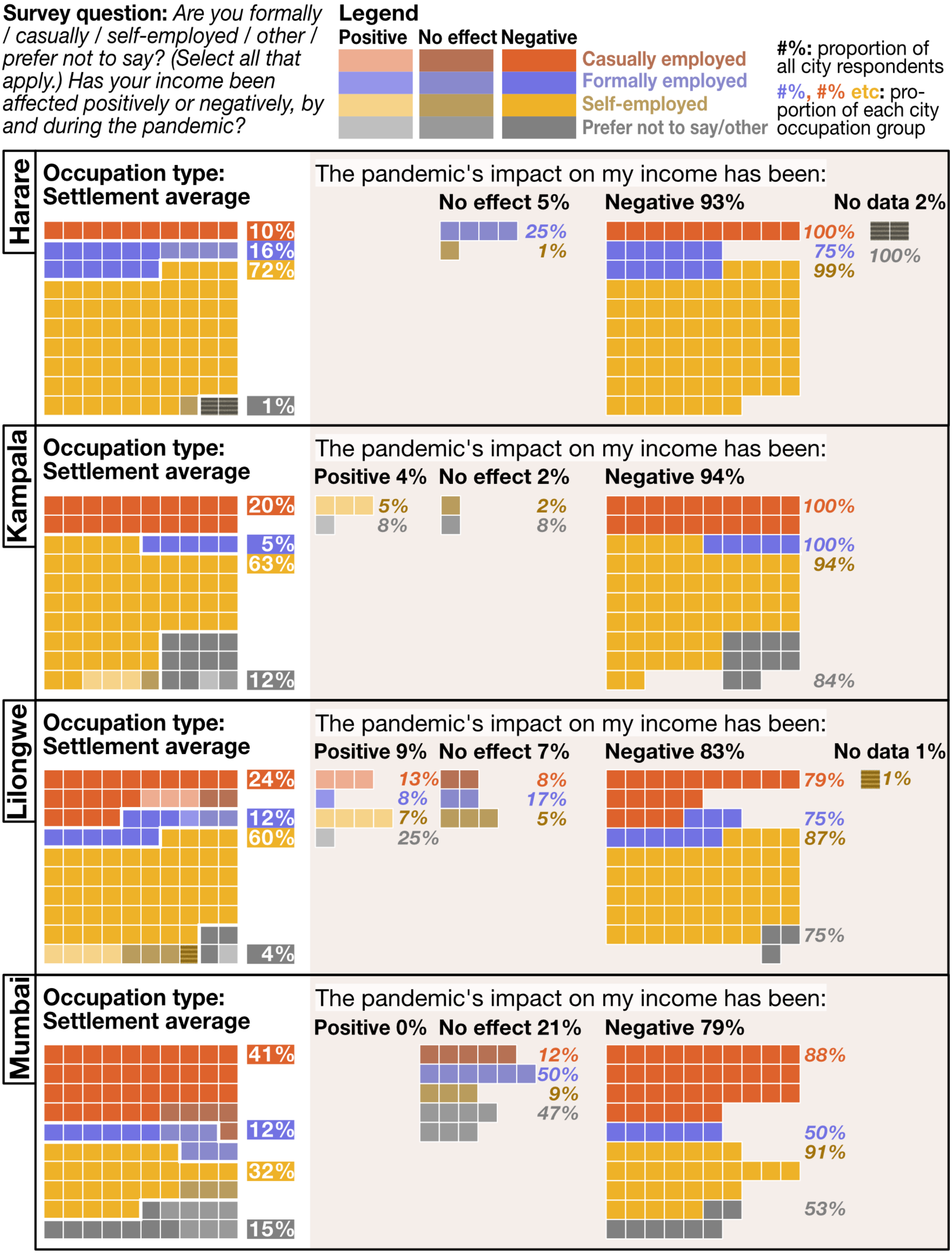

Figure S5: Current lockdown and curfew situation (survey 1 and 6 data; all-settlement average and disaggregated by settlement)

Survey question: *In the past week, has there been an enforced lockdown or curfew in your settlement? What time does the curfew start and end?*

Legend
- Lockdown
- Both lockdown and curfew
- Curfew
- Neither lockdown nor curfew
- Don't know

Notes: Curfew times show median response. Curfew times only shown when a majority of respondents reported curfew.

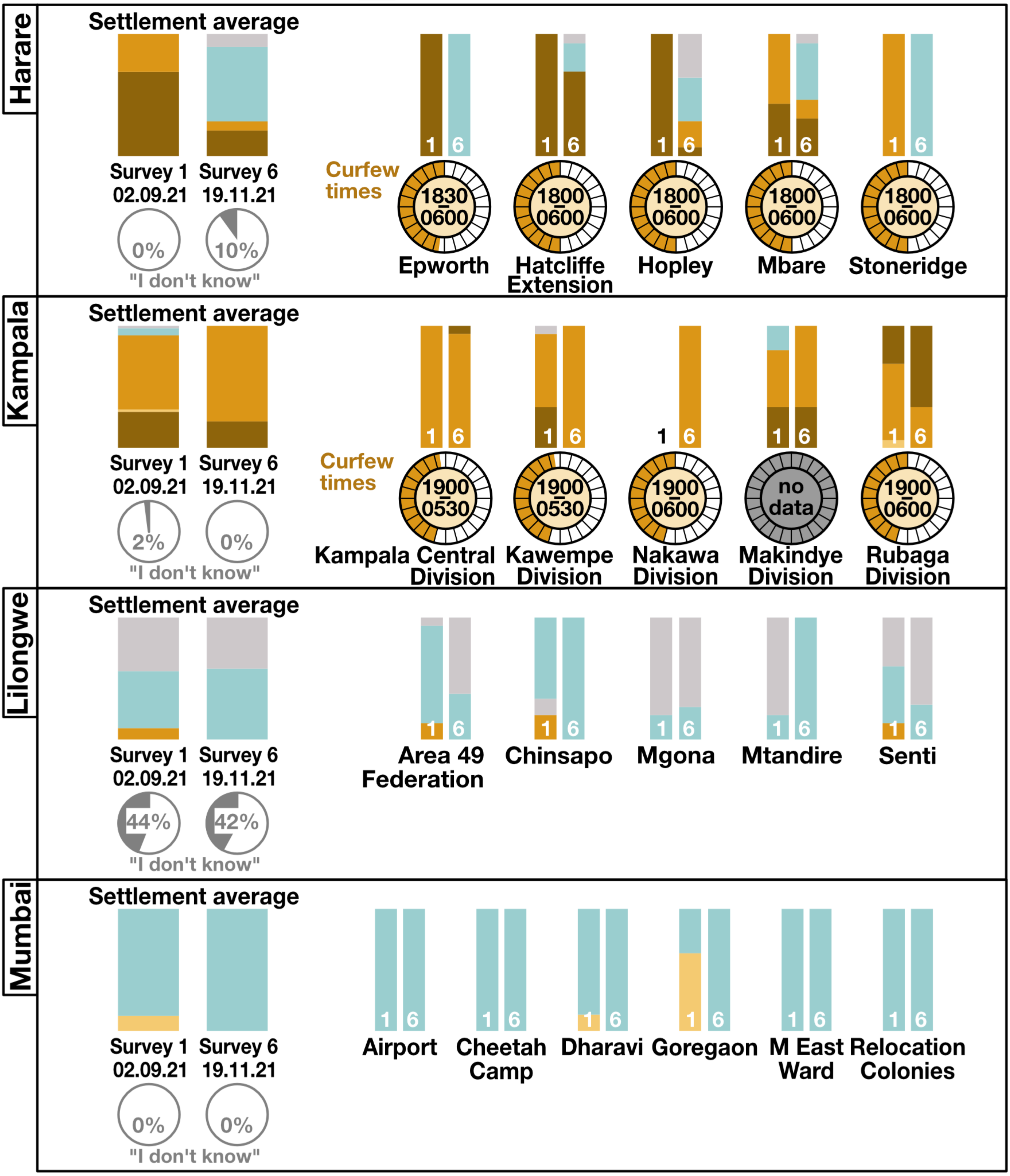

Figure S6: Is a rollout happening? (survey 1–6 data; all-settlement average, by city; disaggregated by settlement)

Survey question: *In the last 2 weeks, has any kind of official Covid-19 vaccine rollout been taking place in your town/city?*

Is the rollout available and accessible to people living in your settlement (regardless of where vaccination is taking place)?

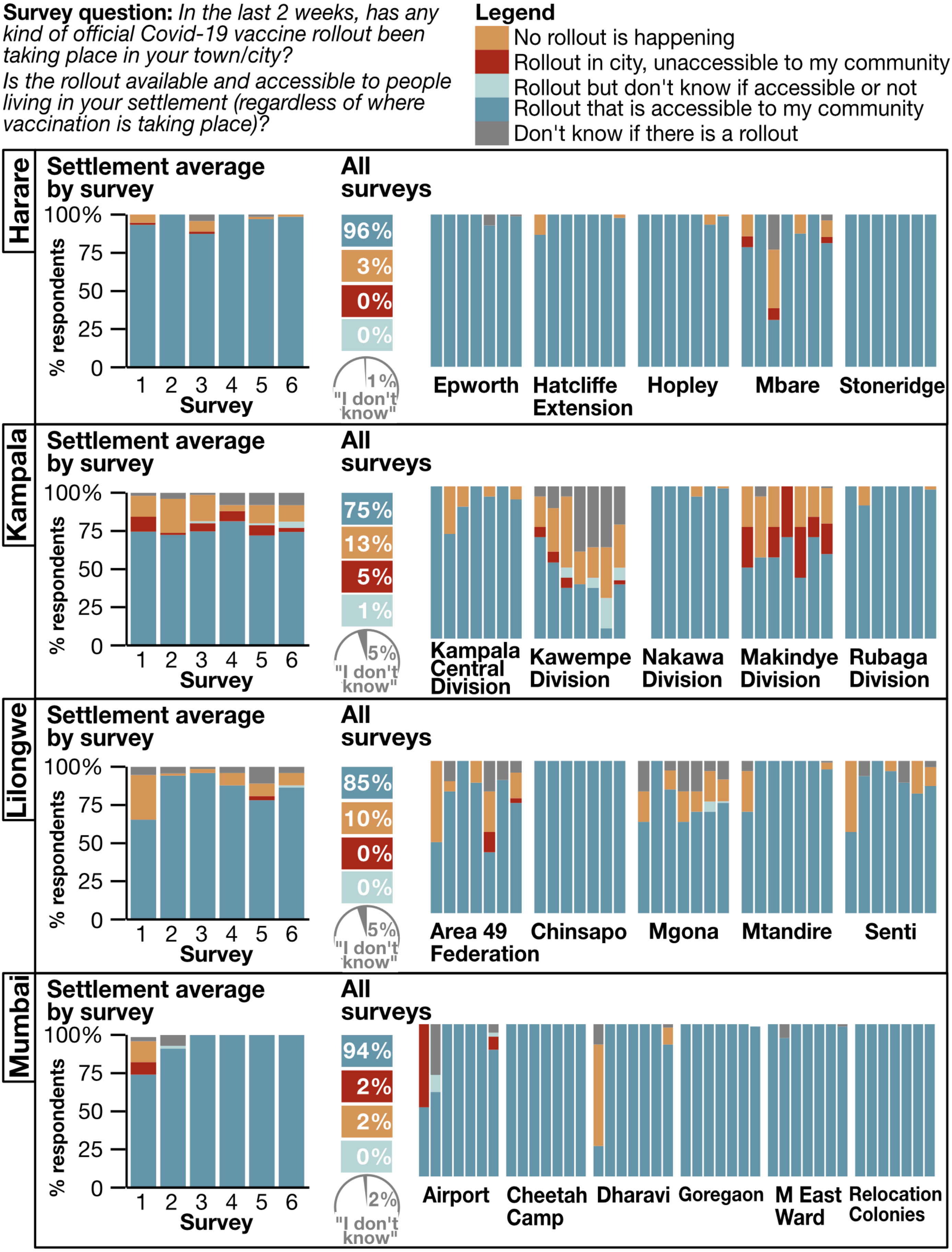

Supplement: sj-pdf-1-eau-10.1177_09562478221149876 – Supplemental material for COVID-19 vaccine rollout: data from informal settlements in Harare, Kampala, Lilongwe and Mumbai [file sj-pdf-1-eau-10.1177_09562478221149876.pdf]
